# Buried Interfaces in Organic Photocathodes for H_2_ Evolution: Fermi-Level Pinning and Recombination

**DOI:** 10.1021/acsami.6c08638

**Published:** 2026-06-25

**Authors:** Eui Hyun Suh, Michel De Keersmaecker, Ratul Mitra Thakur, Bo Dong, Tianquan Lian, Neal R. Armstrong, Erin L. Ratcliff

**Affiliations:** † School of Materials Science and Engineering, 1372 Georgia Institute of Technology, Atlanta, Georgia 30332, United States; ‡ Department of Chemistry, 6572 University of Pennsylvania, Philadelphia, Pennsylvania 19104, United States; § Department of Chemistry and Biochemistry, 8041 University of Arizona, Tucson, Arizona 85721, United States; ∥ School of Chemistry and Biochemistry, Georgia Institute of Technology, Atlanta, Georgia 30332, United States

**Keywords:** organic photocathodes, solar-to-hydrogen conversion, interfacial engineering, recombination mechanisms, self-assembled monolayers, buried interfaces, trap passivation

## Abstract

Herein, we demonstrate how Fermi-level pinning at buried contacts impacts solar fuel generation in all-polymer photocathodes by systematically comparing the effects of work function, hydroxyl coverage, and hydrogen evolution using chemically modified indium tin oxide (ITO) supports. Photovoltages and net photocathode performance are improved when the ITO is passivated using phosphonic acids, independent of work function, suggesting that the passivation reduces Fermi-level pinning at the buried interface arising from blended heterojunction interactions with surface metal hydroxyls. Transient photovoltage decay reveals differences in recombination mechanisms, supported by light intensity-dependent measurements. Briefly, nonpassivated, hydrophilic contacts exhibit trap-assisted recombination, while passivated, hydrophobic contacts follow bimolecular recombination. We then investigate changes in electroactivity of hole-transfer processes as a function of scan rate and repetitive cycling using a diffusion-controlled molecular redox probe, analogous to a hole-only device achieved via the electrolyte. The nonpassivated buried contacts exhibit higher overpotentials for oxidation, indicative of hole injection/extraction barriers. We observe irreversible electron transfer via the hole-transport level of the blended heterojunction and a strong cycle dependence, consistent with changes in the hole trap state density. Passivation results in more reversible redox behaviors, consistent with more Ohmic-like contacts. Collectively, these results provide context toward the realization of durable organic photoelectrodes with optimized photovoltages and net solar-to-hydrogen conversion efficiencies via fundamental understanding of the rates of carrier generation, recombination, and transport in high-dielectric aqueous environments and opportunities to characterize buried interfaces under device-relevant electric fields.

## Graphical abstract

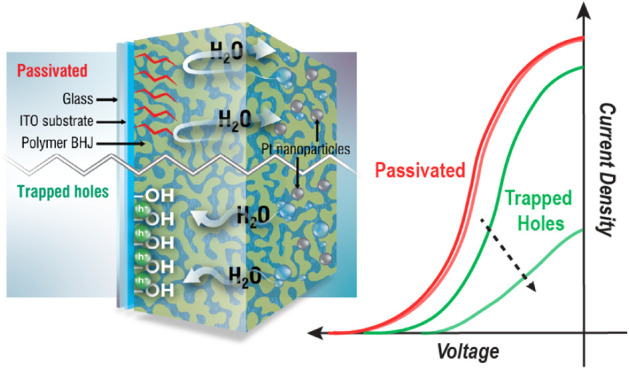

## Introduction

Photoelectrodes convert sunlight into chemical fuels (e.g., H_2_) under highly acidic or alkaline conditions through complex pathways of photocarrier generation, recombination, and extraction, where the latter occurs via redox reactions involving kinetics and mass transport. Conducting polymer semiconductors are promising photoelectrode materials due to their controllable optical properties, good solar spectrum overlap, compatibility with large-area devices, synthetic tunability of redox properties, and long-term feasibility of including catalytic sites (i.e., codesign).^1^ From a fundamental perspective, aqueous electrolyte ingress into the polymer photojunction provides a unique *operando* environment for consideration and is distinct from electronic carrier (photo)­physics studied in other solid-state organic (opto)­electronic devices due to the high dielectric environments imposed by the electrolyte.^2–4^ For example, mixed ionic-electronic conduction, accompanied by dynamic reversible swelling, could yield three-dimensional matrices of reaction sites as a route for high solar-to-hydrogen (STH) conversion efficiency and durable organic photoelectrodes.^5,6^ However, water ingress can also result in parasitic (photo)­electrochemical reactions with electrodes and charge transport layers (e.g., MoO_*x*_, WO_*x*_, SnO_*x*_, and PEDOT:PSS)^7–10^ and should be given further consideration in the context of the durability of organic photoelectrodes. Despite its importance, few studies have focused on investigating and characterizing the effects of electrolyte ingress on charge transfer in organic photoelectrodes.^10–12^

Herein, we investigate two main parasitic recombination pathways for organic photoelectrodes using a model blended heterojunction (BHJ) composed of a polymeric donor and acceptor. Our prior efforts have demonstrated small fractions of water uptake in our BHJ, with an increase in net junction photoconductivity, although contact effects were not considered.^13^ In this work, we show that the net effects of the contacts are not mutually exclusive but rather intimately connected. First, under light bias, the built-in voltage is derived from the quasi-Fermi-level splitting of holes and electrons; contact surface states can lead to Fermi-level pinning of the BHJ charge transport levels (i.e., mismatched energy cascades for hole and electron transport), resulting in reduced built-in bias via traps. Second, the functional groups and defects on metal oxide surfaces are electrochemically reactive in water and can also promote trap sites in various electronic, electrochemical, and photocatalytic applications.^14–16^ The prevalence of hydroxides on so many different metal oxides also makes it challenging to find appropriate materials for electrodes and charge transport layers to operate organic photoelectrodes in harsh environments. Given that the electroactivities of semiconductor materials are dependent on surface states,^17,18^ chemical treatments and/or passivation of buried metal oxide interfaces are a clear opportunity to enhance the lifespans of organic photoelectrodes.

Indium tin oxide (ITO) electrode supports for hole extraction have been widely used in both photoelectrochemical and photovoltaic platforms, but often require modification with interlayer materials.^9,19^ To control work function (WF) and decouple the contact surface from the organic active layer, we compare chemical surface treatments and self-assembled monolayer (SAM) modifications—specifically focusing on phosphonic acids as a straightforward approach.^20,21^ We demonstrate that work function is not the dominant mechanism, where reasonable device performance is achievable when Ohmic contacts are created through ITO passivation. Using transient photovoltage and light intensity-dependent analyses, we indicate clear differences in recombination mechanisms. Passivated contacts exhibit bimolecular recombination, while nonpassivated ones are more trap-assisted (monomolecular). We then use a redox probe to change our rectifying photoelectrodes into hole-only devices, where the redox probe acts as a hole-harvesting top contact. Passivation of the buried ITO surfaces yields reversible redox behavior consistent with an Ohmic contact (again, independent of work function); alternatively, the nonpassivated contacts exhibit hole injection/extraction barriers and irreversible redox behaviors. Coulometric quantification of charge transfer via cyclic voltammetry as a function of scan rate and cycling demonstrates that hole injection/extraction barriers at nonpassivated contacts correlate directly with the decreased photovoltages observed in complete devices and can change under *operando* conditions, indicative of ongoing redox reactions at buried interfaces.

Collectively, the presented approach demonstrates a unique opportunity in photoelectrodes that deviate from solid-state devices. Our approach allows for rapid screening and down-selection of dominant effects such as work function (WF), hydroxyl coverage, electroactivities, and nanometer-scale electrical property heterogeneity on the performances and durability of photocathodes for H_2_ evolution. Half-cells can be readily implemented to elucidate buried contact effects through transport-coupled redox behaviors, both in light and in the dark. Caution should be exerted when considering material selection, especially in efforts to create three-dimensional architectures and hierarchical assemblies. Solar-to-hydrogen (STH) conversion and lifespans of organic photoelectrodes (STH < 4% for a few hours) still lag far behind industrial requirements (STH > 10% for 10 years).^22,23^ Passivation of buried electrodes (or charge transport layers) will be an essential strategy in organic (photo)­electrochemical and bioelectronic platforms.

## Experimental Section

The Supporting Information section contains detailed descriptions of experimental parameters including chemical descriptions of the materials used for the studies, procedures for thin-film construction and characterization, surface treatments, Pt nanoparticle deposition, and device fabrication and testing. In addition, there are detailed explanations of the characterization procedures including cyclic voltammetry, ultraviolet photoelectron spectroscopy, X-ray photoelectron spectroscopy, contact angle measurements, UV–vis-NIR spectroscopy and spectroelectrochemistry, transient absorption, open-circuit measurements, quantification of H_2_ production using gas chromatography, and electrochemical quartz crystal microbalance measurements.

Briefly, here we summarize the construction of the photoelectrodes, with additional information provided in the SI sections. PTB7-Th (M0261A4; *M*_w_ = 125,205 g mol^–1^ and PDI = 2.63) and P­(NDI2OD-T2) (M1201A2; *M*_w_ = 125,509 g mol^–1^ and PDI = 2.3) were purchased from Ossila. PTB7-Th and P­(NDI2OD-T2) were dissolved in chlorobenzene (PTB7-Th:P­(NDI2OD-T2) = 2:1 wt/wt; 8 mg mL^–1^) at 50 °C and the solution was stirred at 800 rpm overnight. The blend solution was spin-coated on pretreated ITO at 2000 rpm for 60 s. After spin coating, the films were annealed at 100 °C for 10 min. Pt nanoparticles were deposited by photoelectrochemical reduction in a three-electrode system, as further described in the Supporting Information section, along with device characterizations.

## Results and Discussion

### Photocathode Design and Performance

Figure 1a shows the energy band diagrams of the photojunction and ITO contacts used in this study; further experimental details are provided in the Supporting Information (SI) section. The photojunction consists of a BHJ comprised of the electron donor poly­[4,8-bis­(5-(2-ethylhexyl)­thiophen-2-yl)­benzo­[1,2-b;4,5-b′]­dithiophene-2,6-diyl-*alt*-(4-(2-ethylhexyl)-3-fluoro-thieno­[3,4-*b*]­thiophene-)-2-carboxylate-2–6-diyl] (PTB7-Th) and the prototypical polymer acceptor poly­{[*N*,*N′*-bis­(2-octyldodecyl)­naphthalene-1,4,5,8-bis­(dicarboximide)-2,6-diyl]-*alt*-5,5′-(2,2′-bithiophene)} (P­(NDI2OD-T2)) with chemical structures shown in Figure 1b. The P­(NDI2OD-T2) acceptor was chosen to minimize dissolution or dispersion into aqueous electrolytes that have been observed for molecular acceptors;^3,24^ our vision is to achieve 3-D photoelectrodes, not create buried junctions under barrier layers (i.e., no electrolyte ingress, termed integrated photovoltaics). Demonstrations using alternative BHJs (Figure S1) and contacts (Figure S2) are provided in Supporting Information Note 1. The energetic alignment between both polymers considered herein was determined to be reasonable, as characterized for PTB7-Th and P­(NDI2OD-T2) via cyclic voltammetry (CV) in 0.1 M H_2_SO_4_ aqueous electrolyte (Figure S3), the same electrolyte used for photojunction operation (Supporting Information Note 2).

**Figure 1:**
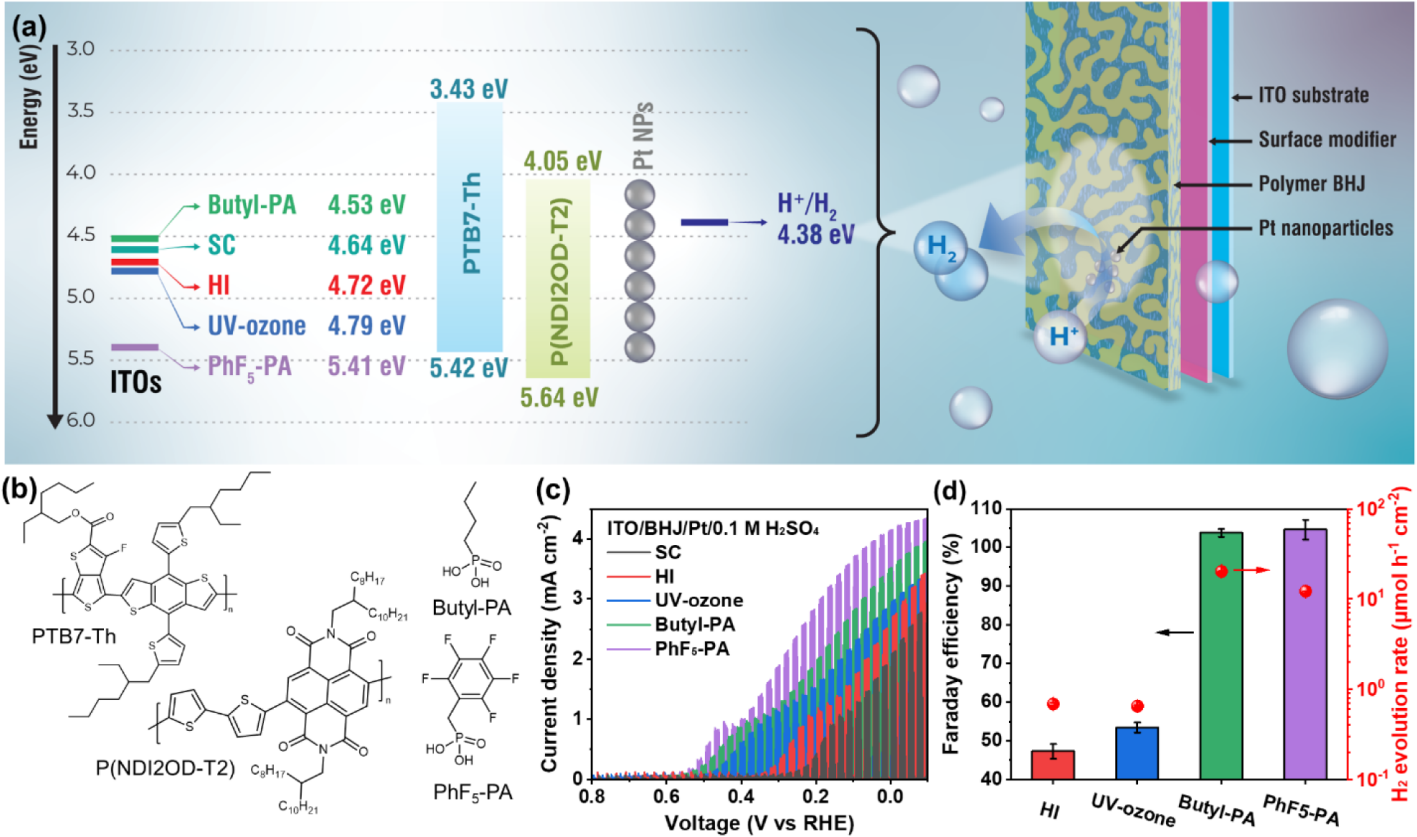
(a) Energy-level diagrams of PTB7-Th, P­(NDI2OD-T2), HER, and surface-treated ITO substrates and schematic illustration of H_2_ evolution in organic photocathodes. (b) Chemical structures of PTB7-Th, P­(NDI2OD-T2), Butyl-PA, and PhF_5_–PA. (c) J–V curves of photocathodes with surface-treated ITOs (ITO/BHJ/Pt) in 0.1 M H_2_SO_4_ under chopped 1 sun illumination. (d) Measured Faraday efficiency and H_2_ evolution rates of the photocathodes during chronoamperometry in an H-cell.

A high-level design guideline in organic optoelectronics is that charge-harvesting (or injecting) contacts should exhibit work functions (WF) that align with the charge transport level of the carrier of interest to minimize extraction losses (i.e., establish Ohmnicity by avoiding Schottky contacts).^19,25^ Schottky contacts often arise through interactions between dangling bonds and/or gap/trap states that “lock” or “pin” the (quasi) Fermi level of the organic material to the contact. Herein, we first investigate if work function alone is a reasonable extrapolation to organic photoelectrodes. We utilized five surface treatments to alter the surface chemistry and WF of the electrode, relative to the transport level of PTB7-Th (5.4 eV in Figure 1a).^25,26^ The WFs of the modified ITO electrodes were measured using shifts in the onset of the secondary electron emission detected by ultraviolet photoelectron spectroscopy (Figure S4). All relevant energy cascade parameters for the photoelectrode are summarized in Figure 1a; electrochemical characterizations are discussed in more detail below. Pt nanoparticle (NP) catalysts with an average radius of ∼90 nm (assessed by atomic force microscopy in Figure S5 in Supporting Information Note 3) were photoelectrochemically deposited on the BHJ layer under white LED illumination to activate the H_2_ evolution reaction (HER); the chemical potential for proton reduction on Pt in an electrolyte of pH 1 was defined as 0.00 V versus RHE (V_RHE_) and is shown as 4.38 eV in Figure 1.^27^

The HER performance of the five photocathodes (ITO/PTB7-Th:P­(NDI2OD-T2)/Pt) with the different surface-treated ITO substrates was evaluated by linear sweep voltammetry (LSV) in a 0.1 M H_2_SO_4_ aqueous electrolyte (pH 1) under chopped 1 sun illumination at a scan rate of 20 mV s^–1^ (Figure 1c). The H_2_ gas from the five photocathodes was collected for 30 min in an H-cell and quantified by gas chromatography (Figure 1d). The calibration curve is demonstrated in Figure S6. Passivating the buried ITO interface with phosphonic acid self-assembled monolayers (PA-SAMs) yielded the highest H_2_ production rates, independent of work function. Specifically, butyl phosphonic acid (Butyl-PA) and pentafluorobenzyl phosphonic acid (PhF_5_–PA) reached 20.25 ± 0.20 and 12.20 ± 0.02 μmol h^–1^ cm^–2^, respectively, both maintaining Faradaic efficiencies near 100%, despite differing work functions. However, only small amounts of H_2_ gas evolved from photocathodes constructed on Hydroiodic acid (HI) and UV–ozone treated ITO surfaces, which increased measurement errors and resulted in low Faradaic efficiencies of ∼50%. The measured H_2_ evolution rates are comparable to photocatalysts based on organic semiconductor nanoparticles (1.05–28.52 μmol h^–1^ cm^–2^), as summarized in Table S1.^28–36^

In Figure 1c, there are clear contact effects in both the photovoltage (the voltage onset in chopped photocurrent, V_onset_) and the photocurrent at 0.00 V_RHE_ (J_V=0_). The PA-SAM-passivated ITO photocathodes achieved the highest photovoltage and photocurrent, while nonpassivated chemical treatments (UV–ozone, HI, and solvent cleaning) resulted in a consistent efficiency loss due to lower V_onset_ and J_V=0_. This observation is independent of the Pt catalyst, as further justified in Figure S7, where we detect similar photovoltage trends during Pt deposition and the oxygen reduction reaction (ORR) in all ITO/BHJ devices.

Our observation that both photovoltages and photocurrents trend with contact passivation leads us to conclude that recombination and extraction are in direct competition, regardless of the applied potential versus RHE. More specifically, recombination is increased in photoelectrodes (over organic photovoltaics) because charge extraction at the top electrode (here the electrolyte) is slower due to a redox reaction; in slow extraction devices, the impacts of Fermi-level pinning become even more important, effectively yielding insufficient quasi-Fermi-level splitting.^19,37^ Prior reports of PTB7-Th/P­(NDI2OD-T2) photovoltaic cells at 1 sun exhibit photovoltages of ∼0.85 V and short-circuit currents of ∼10 mA cm^–2^.^3,38^ We rationalize that the photocathodes in Figure 1 have lower voltages and current densities than solid-state devices due to two major factors. First, electron transfer rates through the Pt catalysts are dependent on the concentration of reactants (protons undergoing mass transport toward the surface). Changing the pH would thus lower the concentration and therefore the rate of charge transfer.^39^ As a point of comparison, J_V=0_ and applied-bias photoconversion efficiencies (ABPEs) followed the trends with V_onset_ in acidic (pH 1) and neutral (pH ∼7) electrolytes (Supporting Information Note 3; Figure S8; Tables S2 and S3), where for each contact, photocurrents decrease with increasing pH. Again, passivated contacts outperform nonpassivated ones. Second, the charge transfer rate at the Pt is limited by the detachment of H_2_ gas bubbles, which is sporadic in these devices. As shown in Supporting Information Note 4 and Figure S9a, time-dependent photocurrent loss is due to the formation of gas bubbles that block the catalytic active area; when bubbles detach, the photocurrent rapidly rises (i.e., increased area of Pt/electrolyte) and decays again as gas bubbles continue to form and block the catalyst. We hypothesize that the initial current drop cannot be attributed to PA-SAM desorption, as these monolayers remain stable on oxide surfaces in acidic environments (pH <7).^40^ Specifically, both PA-passivated contacts continue to outperform the nonpassivated contacts in chronoamperometry and successive scanning (Figure S9b). Bubble retention is evident in Figure S8c.

We speculate that the improvement is due to a reduction in trap states at the buried interface of ITO/BHJ. Briefly, the composition of most metal oxide surfaces is dominated by hydroxides rather than the stoichiometric oxide, where the hydroxyl coverage is intimately connected to electrochemical reactivity (i.e., the basis for Pourbaix phase diagrams). The correlation of hydroxyls to trap states at buried interfaces is consistent with various electronic, electrochemical, and photocatalytic application reports, which are correlated with the outcome of increasing recombination.^15–17^ We first consider the chemical composition of the near-surface region and supportive evidence for contact-specific stabilities, followed by quantification of the carrier lifetimes and recombination coefficients (i.e., recombination order) in the following sections to support our hypothesis.

### Interface Composition and Water Penetration in Photocathodes

As the metal hydroxyls on the ITO are hypothesized to be connected to Fermi-level pinning, we assessed hydroxyl coverages using O 1s core-level X-ray photoelectron spectroscopy spectra (XPS; Figure 2a) and the surface wettability using water contact angles (Figure 2b). The C 1s, P 2p, F 1s, Sn 3d, and In 3d core-level spectra are summarized in Figure S10. Schematic representations of the near-surface chemistry are shown in Figure 2c based on the XPS analysis in Supporting Information Note 5. Briefly, we determine that these results are consistent with prior reports; interpretations are summarized in more detail in Supporting Information Note 5, including Figure S9; Tables S4 and S5:

- Solvent cleaning (SC) removes some of the adventitious carbon contaminants but does little to electrochemically activate the near-surface (top 10 nm).^41,42^

- HI and UV–ozone treatments remove organic contaminants from the surface. HI etches the top few nanometers of the ITO surface to reveal a highly activated, fresh oxide layer, while UV–ozone treatment forms a hydroxide layer through chemical reactions with atmospheric components (e.g., H_2_O vapor) on the oxygen-rich surface.^42^
- Butyl-PA and PhF_5_–PA treatments modify the surface through bidentate and tridentate bonding with surface hydroxyls to passivate buried ITO/polymer interfaces and suppress electrochemical reactivity^43^ but have very different WFs due to opposing dipole moments (Figure 1a).^19,44,45^ Furthermore, Butyl-PA has a linear alkyl chain forming a denser layer than PhF_5_–PA that includes a bulky phenyl ring, which passivates the ITO surface more effectively.

**Figure 2:**
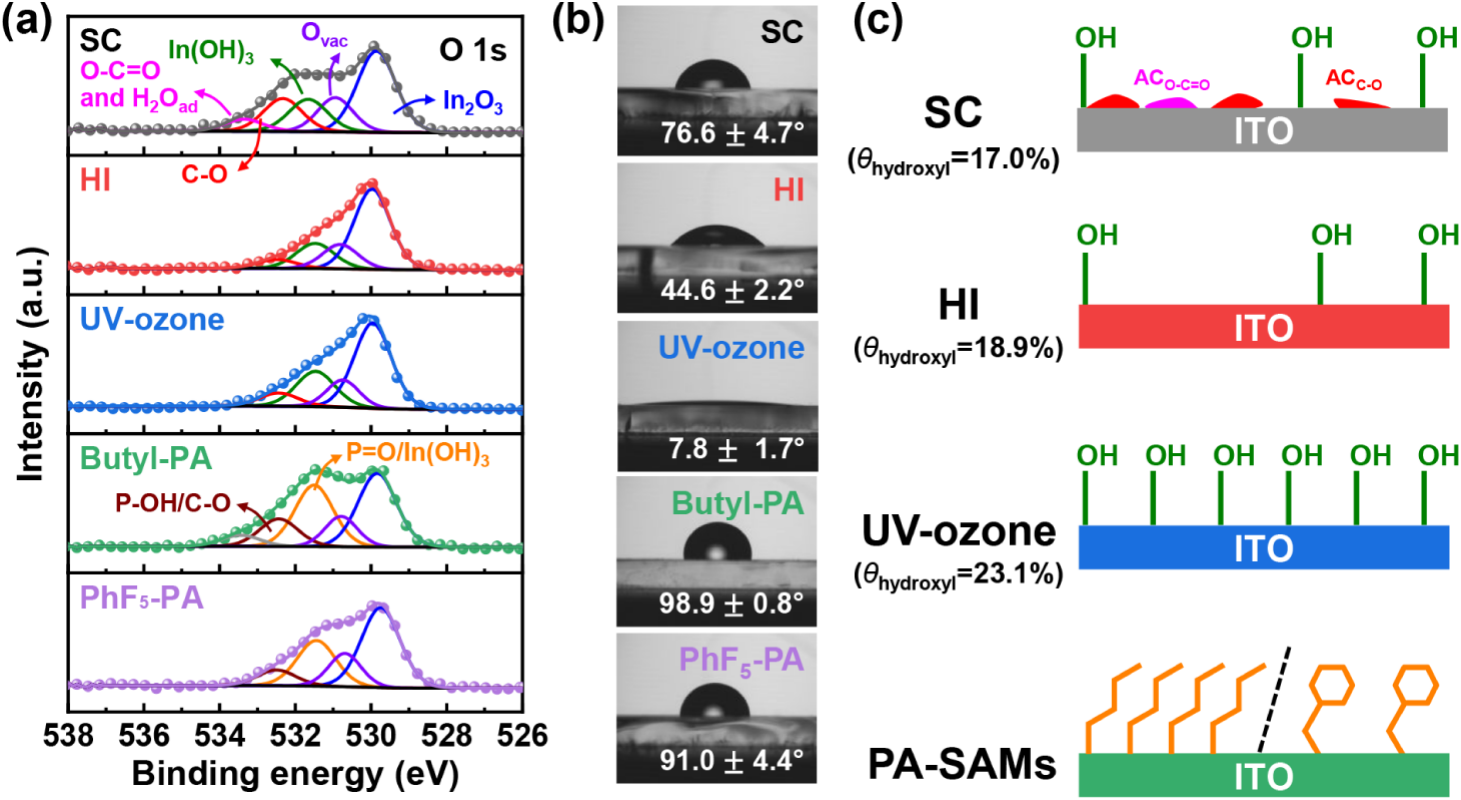
Surface treatments on the ITO substrates are verified by assessing the near-surface region with (a) XPS spectra of the O 1s core levels and (b) water contact angles. (c) Schematic illustration of the ITO surface chemistry, where θ_hydroxyl_ (=Area­(O 1s)_hydroxide_/Area­(O 1s)_total_) is defined as the surface coverage of hydroxyl groups. For the solvent-cleaned (SC) ITO, the surface is contaminated with C–O-containing species shown in pink and red.

Water contact angles of the surface-treated ITO substrates illustrate the hydrophilic/hydrophobic nature of the functional groups defined by XPS (Figure 2b).^46^ Organic contaminants increase the hydrophobicity of solvent-cleaned ITO, while HI etching and UV–ozone treatment reduce the contact angles by eliminating adventitious carbon contaminants. A water contact angle of nearly 0° on UV–ozone-treated ITO is consistent with a dense structure of polar hydroxyl groups. In contrast, PA-SAM-modified ITO substrates demonstrate increased water contact angles, which we attribute to the hydro- and fluorocarbon groups. The hydrophobicity of the PA-SAM-modified ITO introduces a more pinhole-free interface with the BHJ, which reduces parasitic reactions facilitated by bare ITO and surface recombination effects.

Evidence of electrolyte penetration within the BHJ is demonstrated in Supporting Information Note 6 based on electrochemical quartz crystal microbalance (EQCM) measurements (Figure S11). Voltammetric scans within the nonfaradaic window in a 0.1 M H_2_SO_4_ reveal a small frequency change in Figure S10, supported by our prior results of water ingress using X-ray reflectivity.^13^ Additional corroborative evidence for electrolyte penetration impacting the photoelectrodes via contact effects is given in Figures S12–S15. First, we increased the BHJ thickness from approximately 40 to 130 nm by increasing the donor–acceptor concentrations (Figure S12); if water is diffusing in, increasing thickness should reduce redox reactions at first due to longer diffusion paths. The nonpassivated contact showed a major loss in normalized photocurrent with each progressive scan (Figure S12), with a strong dependence on the thickness of the junction (Figure S13e). Alternatively, the passivated contact showed minimal loss in normalized photocurrent with repeated scanning, regardless of the BHJ thickness (Figure S13f). Second, we removed residual water via exposure to vacuum (Figure S14), showing an immediate improvement in the rectification and stability of nonpassivated ITO photocathodes. This treatment was not necessary on the passivated contacts, as discussed for Figure S8b. Third, we held the devices at a bias near 0.0 V_RHE_ for an extended period of time. We then repeated the linear sweep. Nonpassivated contacts again showed major losses, consistent with ongoing water ingress + redox reactions, while performance for passivated contacts was retained (Figure S15). We next focus on recombination effects due to contacts.

### Buried ITO/BHJ Interfaces and Their Influence on Recombination Mechanisms

The photocathodes with treated buried interfaces are characterized via transient open-circuit potential (OCP) and transient J_V=0_ measurements under chopped 1 sun illumination to assess their recombination mechanisms (Figure 3a). The slope of the OCP decay when the light is turned off can be used to determine the carrier lifetime as^47^

1 $$\tau_{n}=-\frac{k_{B}T}{q}{\left(\frac{d\mathrm{OCP}}{dt}\right)}^{-1}$$

where *k*_B_ is the Boltzmann constant, *T* is the absolute temperature, *q* is the unit charge, and *t* is the time. Figure S16a shows an example of an OCP versus time transient, and Figure S16b shows carrier lifetimes for each of the contacts. The photocathodes with PA-SAM-treated ITO electrodes demonstrate much shorter lifetimes τ_n_ (<1 ms) compared to the photocathodes with nonpassivated contacts. Mechanisms of recombination are described by the β parameter that can be derived from the OCP–*t* curves in Figure 3b and eq 2.^47^

2 $$\beta =1+\frac{k_{B}T}{q}\frac{d\ln{\tau_{n}}^{-1}}{d\mathrm{OCP}}$$

**Figure 3:**
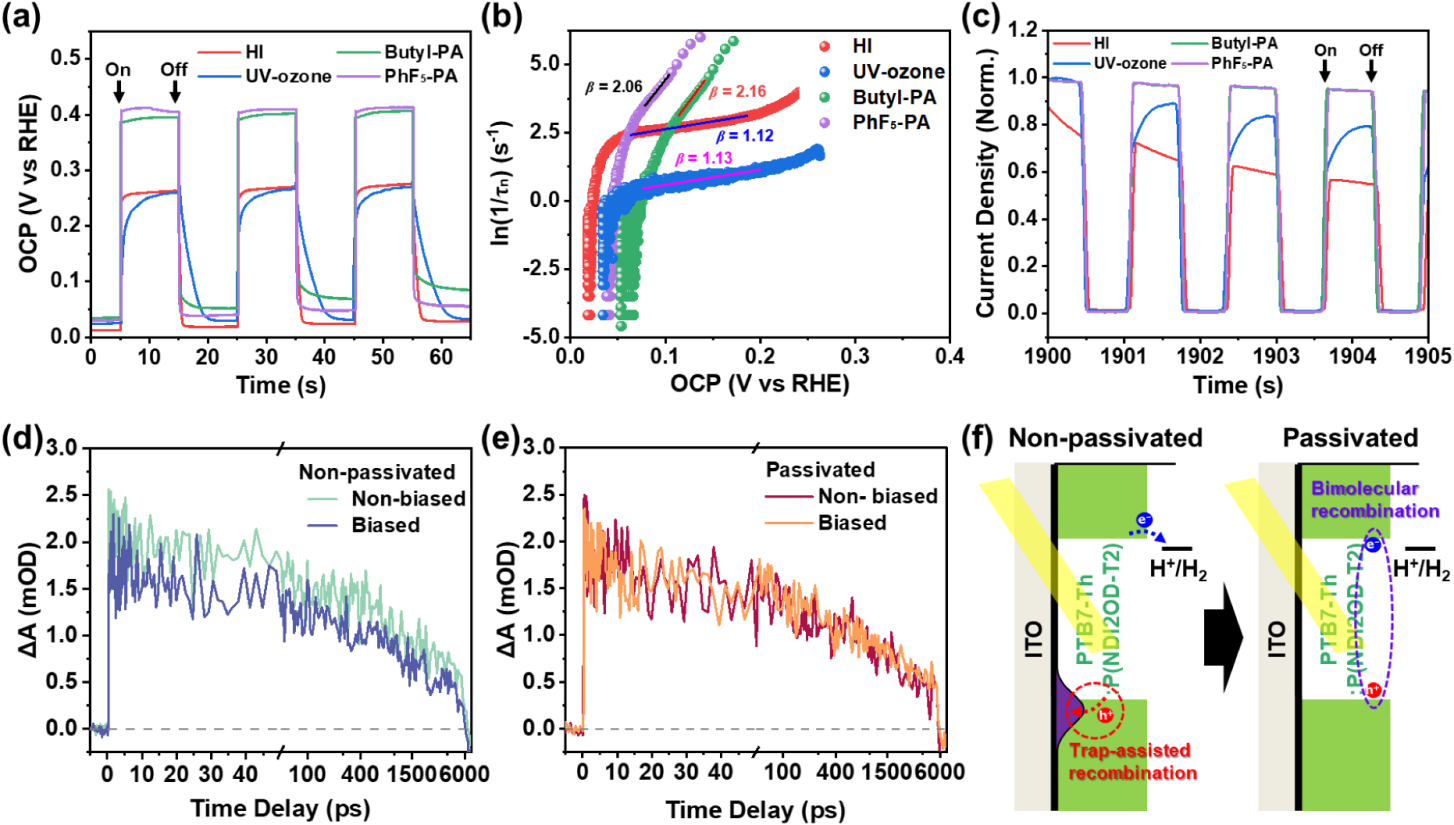
(a) Transient open-circuit potential (OCP) curves of photocathodes (ITO/BHJ/Pt) under 1 sun illumination and (b) respective time constant profiles as a function of OCP which have been extracted from the transient OCP decay at 15 s. (c) Transient photocurrents of the photocathodes under 1 sun at 1900–1905 s. Transient absorption kinetics curves of (d) nonpassivated (UV–ozone) and (e) passivated (Butyl-PA) photocathodes before and after five LSV scans in aqueous 0.1 M H_2_SO_4_ electrolyte. The photocathodes were pumped at 650 nm (fluence = 5.76 μW) and probed at 1100 nm. (f) A schematic of recombination mechanisms in the organic photocathodes with nonpassivated and passivated ITO electrodes.

A β value of 1 is indicative of trap-assisted recombination, while a β value approaching 2 reflects more radiative or bimolecular recombination.^48^ From the calculated β values in Figure 3b, the nonpassivated HI and UV–ozone-treated photocathodes (β = 1.12 ± 0.01 and 1.13 ± 0.01, respectively) follow a trap-assisted recombination process, while passivation via Butyl-PA or PhF_5_–PA-treated photocathodes (β = 2.16 ± 0.01 and 2.06 ± 0.01, respectively) is defined by bimolecular recombination. Similar trends are demonstrated in the transient photocurrent (J_V=0_) curves, where nonpassivated photocathodes show a delayed rise in photocurrent, a phenomenon that is attributed to slow trap filling upon light illumination (Figure 3c).^49^ We note that the transient J_V=0_ of Butyl-PA and PhF_5_–PA-treated photocathodes responds more rapidly upon changing light conditions, which implies that no hole trap states are present at the ITO/BHJ junction.^50^

A complementary measurement for assessing recombination mechanisms is to consider the direct proportionality between light intensity and the initial charge carrier density (*I* ∝ *n*_0_), the latter assessed via the current density at 0 V_RHE_ (J_V=0_). More specifically, deviation of the slope of J_V=0_ versus light intensity from a value of one can be used as an independent indicator of deviation from bimolecular to trap-assisted recombination.^51,52^ The nonpassivated buried contact demonstrates a slope of 0.77 in Figure S17a, weighted to poorer performance (i.e., higher rates of recombination) at higher light intensities, while the device constructed on the passivated ITO contact in Figure S17b is closer to 1.0. Recombination effects are amplified in the transient photocurrents; an example is shown in Figure 3c after approximately 1900 s of light modulation. Devices using passivated contacts retain current density, whereas the non-passivated ones show slow rise times in photocurrent when the light is turned on. Figure S18 shows the transient photocurrent effects over 3600 s, including cases where the electrolyte is removed and refilled. When the electrolyte is removed and refilled, nonpassivated contacts start to recover (consistent with results from Figure S13) and then fail again, indicative of a trap mechanism derived from water ingress.

We also considered the possibility that passivated versus nonpassivated contacts could impact free carrier generation rates. For reference, Figure S19 shows comparative transient absorption spectra before and after biasing for the two photocathodes. Figures 3d and 3e provide transient absorption kinetics of PTB7-Th exciton and hole polaron signals, probed at 1100 nm,^53^ confirming that the rate of carrier generation in the BHJ was preserved after LSV scans were taken using photocathodes with passivated ITO electrodes. In contrast, the corresponding signals were reduced in the biased nonpassivated ITO photocathode, which is consistent with faster decaying polaron signals by trap states. Another way to assess stability is given by a comparison between electrochemical impedance spectroscopy (EIS) measurements at 0.25 V_RHE_, the potential at which maximum ABPE is obtained. We conducted EIS before and after the LSV scans, as detailed in Supporting Information Note 7 (Figures S20 and S21; Table S6) for devices with passivated and nonpassivated contacts. The nonpassivated photocathode shows an increase in the double-layer capacitance at the ITO/BHJ interface after multiple LSV scans (circuit diagram in Figure S20; *C*_CT_ in Table S6)^11,12^ and simultaneously exhibits a clear indication of mass transport effects (i.e., Warburg behavior at low frequencies); Warburg elements were not observed in the passivated photocathode. These results support that electrolyte ingress leads to unwanted interactions at nonpassivated metal oxide contacts ultimately increasing the number of hole traps at the ITO/BHJ interface. Collectively, we propose that the hydroxyl groups on nonpassivated contacts act as hole traps that cause trap-assisted recombination, while passivation eliminates these traps so that bimolecular recombination becomes dominant, as summarized in Figure 3f. The net effect is that nonpassivated surfaces lead to Fermi-level pinning and Schottky contacts, while passivation promotes more Ohmic contacts. This effect can be revealed in hole-only devices.

### Hole-Only Devices Using Chronocoulometry with a Redox Couple

Hole injection and extraction through the buried ITO/BHJ interfaces can be assessed using chronocoulometry to track electron transfer to a molecular redox probe; similar approaches have been used to quantify defect states in lead halide perovskite materials^54^ and to investigate density of states (DOS) distributions of semiconductors and trap states.^55–57^ In this case, the oxidation and reduction of water-soluble ferrocenemethanol (FcOH) are measured in an acidic aqueous electrolyte. Figure 4 shows a combination of schematics (a–c) and CV data (d–f) to clarify charge movement in the anodic and cathodic scans. Our materials stack is characterized in the dark and, thus, can be considered analogous to a hole-only device via which we monitor hole injection/extraction and transport through the BHJ; this structure also includes an Ag/AgCl reference electrode to provide an energy scale calibration. Briefly, by tracking the current in the oxidation waves of the CV, we can focus on the hole injection process from the ITO, through the blend, to oxidize FcOH to FcOH^+^ in the electrolyte. The reverse wave tells us about hole extraction, whereby FcOH^+^ is reduced and the hole travels through the blend and is collected by the ITO electrode. We have restricted the analysis to a comparison between nonpassivated ITO and passivated ITO for simplicity; an expanded discussion of the other electrodes is provided in Supporting Information Note 8.

**Figure 4:**
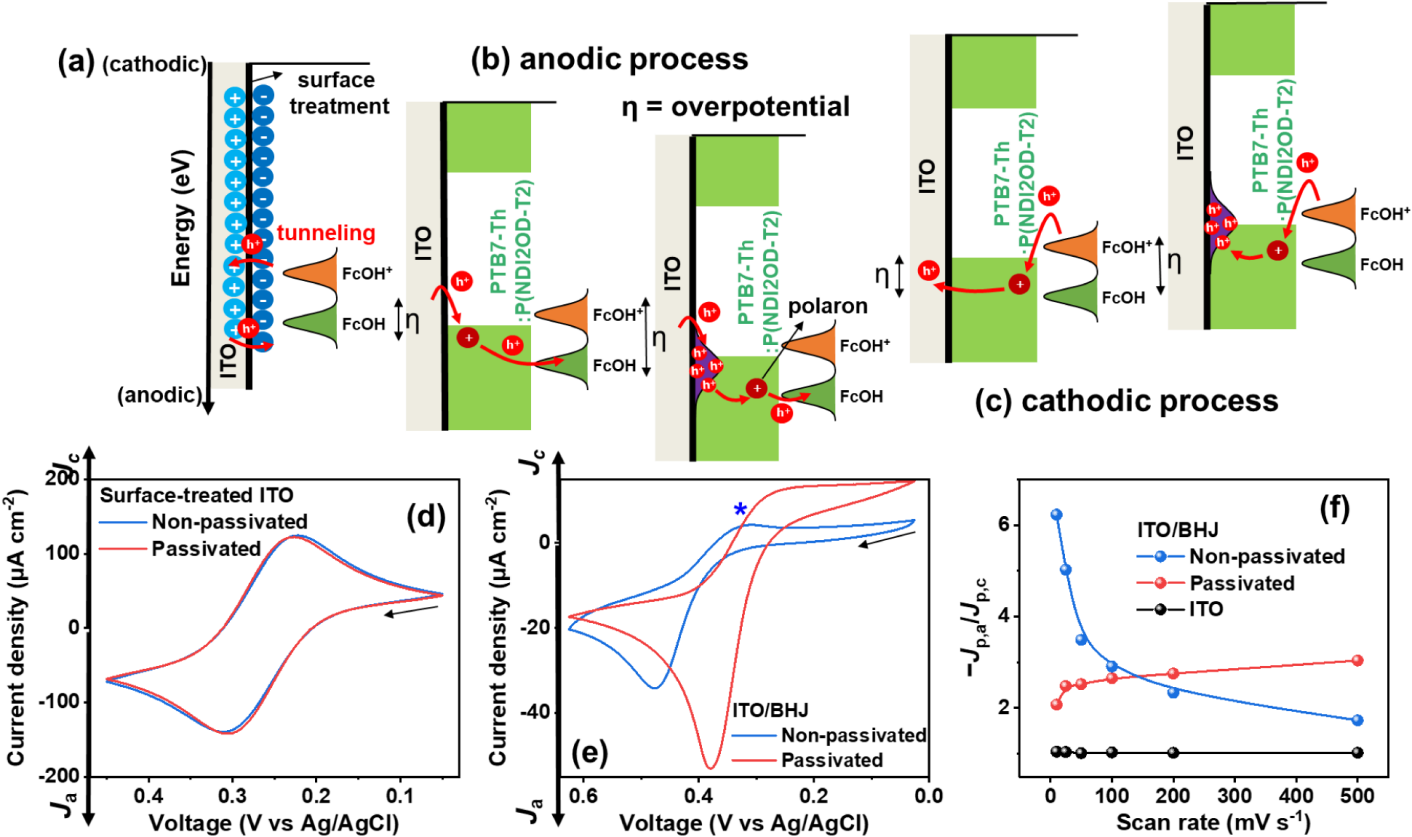
Schematic for the (a) tunneling process on surface-treated ITO, (b) anodic, and (c) cathodic hole transfer processes for ITO/BHJ photocathodes in the presence of a hole-capturing redox probe with and without surface states at the ITO/BHJ interface. (d) CV curves of surface-treated ITO and (e) nonpassivated (UV–ozone) and passivated (PhF_5_–PA) hole-only electrodes (ITO/BHJ) in aqueous 1 mM FcOH + 0.1 M H_2_SO_4_ electrolyte under dark conditions. The direction of oxidation is provided, and all CVs were collected at a scan rate of 50 mV s^–1^. (f) Scan rate dependence of the peak anodic current and peak cathodic current densities of the hole transfer process for surface-treated ITO electrodes and ITO/BHJ electrodes.

First, when a metal oxide (i.e., ITO) is brought into contact with an electrolyte, the Fermi-level pins the standard potential of the added redox probe, as illustrated in Figure 4a; band bending is expected to be minimal as ITO is degenerately doped. Alternatively, for the organic semiconductor films in Figure 4b,c, the oxidation and reduction of FcOH/FcOH^+^ are dependent on the available electrochemically active DOS of the BHJ, assuming the Marcus–Gerischer model for semiconductors.^58–60^ An overpotential (η) in either the forward or backward linear sweeps on the polymer electrodes, relative to the *E*_1/2_ of the reversible FcOH/FcOH^+^ probe behavior on ITO, reflects a combination of BHJ DOS and Schottky contact effects, all relative to the charge transfer states of the redox probe. Figure 4b summarizes two different energetically activated hole-injection processes across the junction, where the hole is immediately captured by the redox probe to drive the oxidation of FcOH to FcOH^+^. The left side of Figure 4b shows the ideal case and the right side shows a schematic of the trap states at the ITO/BHJ interface due to Fermi-level pinning. In Figure 4b (right side), the interfacial traps must first be filled with holes before polarons can transport through the BHJ, impeding hole injection for FcOH oxidation. The cathodic process (i.e., reverse reaction) in Figure 4c is characterized by the reduction of the redox probe, with the harvested hole traveling through the BHJ and then being injected back into the ITO electrode. Again, the ideal (left) and trap-assisted (right) schematics are presented. Changes in reversibility are discussed further below, with considerations of scan rate (Figure 4) and repetitive cycling (Figure 5).

**Figure 5:**
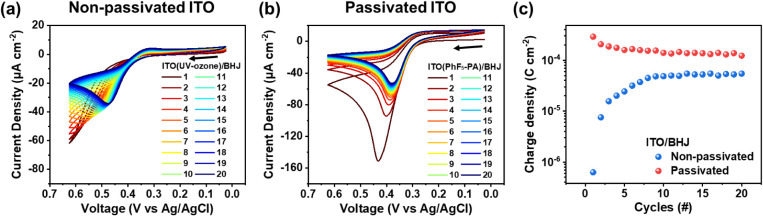
CV scans of (a) nonpassivated (UV–ozone) and (b) passivated (PhF_5_–PA) hole-only electrodes (ITO/BHJ) in 1 mM FcOH + 0.1 M H_2_SO_4_ electrolyte at a scan rate of 50 mV s^–1^. The black arrow represents the direction of the oxidation scan. (c) Charge densities of FcOH oxidation peaks in the CV scans as a function of cycle number.

Figure 4d,e shows the CV for the treated ITO surfaces and with the BHJ, respectively. In Figure 4d, with no BHJ, the two electrodes yield equivalent CV behavior at 50 mV s^–1^, exhibiting relatively slow but (quasi)­reversible charge transfer with a peak separation of 78 mV centered at 0.27 V versus Ag/AgCl (V_Ag/AgCl_). In Figure 4e, we observed that the electrochemical reversibility and overpotentials are greatly affected by the buried interfaces. The nonpassivated ITO/BHJ electrode exhibits an oxidation peak potential shifted to approximately 0.48 V_Ag/AgCl_, over 170 mV more anodic than the ITO electrodes without the BHJ. The PA-SAM-modified ITO/BHJ electrode has a lower overpotential for FcOH oxidation, with a peak potential at approximately 0.38 V_Ag/AgCl_ and an onset potential at 0.30 V_Ag/AgCl_. Both electrodes have lower net current densities than the treated ITO surfaces, reflecting impeded charge transfer, relative to a metal-like continuum of states of the ITO. The decreased current densities and overpotentials are expected, consistent with semiconductor electrochemistry. First, polymers have low DOS relative to metals, so unless charge transfer is mass transport-limited (rather than kinetically), currents will be lower on polymers. Overpotentials are required due to the energy distribution of states; a semiconductor can only participate in charge transfer if a state is available. As the oxidation potentials of the PTB7-Th donor materials are more anodic than the FcOH oxidation (i.e, harder to oxidize), oxidation of FcOH only occurs once a hole has been injected by the ITO and travels through the BHJ to the interface (Figure 4b). Shifts in FcOH for the two contacts thus directly indicate hole injection barriers. More specifically, the nonpassivated ITO surface has a greater hole injection barrier (i.e., requires a more anodic overpotential of 170 mV to oxidize FcOH). We do not expect the density of states of the PTB7 to change significantly.

The reverse cathodic wave scan follows the schematic shown in Figure 4c. The PA-SAM-passivated ITO/BHJ electrode exhibits higher net current densities for FcOH^+^ reduction over the nonpassivated ITO/BHJ electrode (Figure S22); neither semiconductor junction demonstrates the same magnitude as the respective current densities for FcOH oxidation or FcOH^+^ reduction on ITO, indicating some irreversibility. The reversibility of a reaction can be qualitatively assessed by considering the ratio of the peak anodic current density (J_p,a_) relative to the peak cathodic current density (J_p,c_) as a function of scan rate. Raw data for the two contacts are presented in Figure S23 and peak ratios are shown in Figure 4f. For the ITO-only surface, the anodic and cathodic peaks have a ratio of ∼1 for the scan rates considered in Figure 4f (black points), indicating reasonable reversibility. Alternatively, the BHJ systems reflect quasi-reversible behavior for the passivated ITO contact (moderate scan rate dependence) and irreversible behavior for the nonpassivated contact (strong scan rate dependence). Importantly, the nonpassivated ITO buried interface shows the greatest asymmetry in the anodic and cathodic current density ratio at slower scan rates.

Nonpassivated and passivated ITO/BHJ/FcOH hole-only devices were then CV scanned for up to 20 cycles (Figure 5a,b, respectively); Figure S24 shows data for other contact/BHJ hole-only devices. In Figure 5a, the nonpassivated ITO electrode shows two key regions in the oxidation wave as a function of scan number. During the early scans, anodic current densities are highest near 0.6 V; after approximately 8 cycles, peak current densities are at 0.45 V and reflective of expected redox probe behaviors (clear duck shape). Similar effects were observed for HI-treated ITO/BHJ and SC ITO/BHJ electrodes (Figure S23). As a result, we postulate that the early scans are dominated by traps, which could be due to small amounts of water ingress as observed in the EQCM data (Figure S10).

Integration of the areas of the anodic and cathodic waves allowed us to quantify the hole concentration participating in the redox reactions (i.e., *Q*_anodic_ and *Q*_cathodic_). A schematic for the two waves is shown in Figure S25. Figure 5c summarizes *Q*_anodic_ for passivated and nonpassivated contacts as a function of scan number. We readily observe that as we continue to scan the nonpassivated hole-only devices, we increase the number of holes that can participate, consistent with filling the traps. Likewise, in Figure S26, turning on the light (i.e., increasing the photoconductivity of the hole-only devices) increases the reversibility of the reaction, consistent with photogenerated carriers filling traps.

## Conclusions

In summary, we have demonstrated that extrapolation of contact effects from organic photovoltaics to organic photoelectrodes should be undertaken with caution because of a combination of new redox chemistries and changes in dielectric constants. Metal hydroxides, which are representative electroactive sites, were correlated with hole trap states at buried ITO-polymer junctions by electrochemical reactions that demonstrated effects on recombination. The ideal scenario is stable Ohmic contacts at the buried junction. Furthermore, our study highlights fundamental semiconductor electrochemistry and insights that can be gained when considering different redox probes, which undergo charge transfer at different potentials, to evaluate the Ohmic nature of the buried interface. More broadly, passivation of buried electrodes (or charge transport layers) will be an essential strategy to utilize electrochemically reactive charge transport layers and electrodes, including oxides, halides, and polymers, for organic photoelectrodes but also for other (photo)­electrochemical systems.

## Supplementary Material



## Notes

The experimental section, device characterizations under various conditions, CV scans, UPS spectra, AFM images, XPS spectra, UV–vis spectra, transient absorbance spectra, carrier lifetime measurements, transient photocurrents, electrochemical impedance data, hole and electron extraction efficiency, and tables (PDF)
