# Supplementary material for: Buried Interfaces in Organic Photocathodes for H2 Evolution: Fermi-Level Pinning and Recombination
Source: ACS Appl Mater Interfaces. 2026 Jun 25;18(26):36831–41. doi: 10.1021/acsami.6c08638 (PMC13352507; doi:10.1021/acsami.6c08638)
Supplement: Supplementary file 1 [file am6c08638_si_001.pdf]

Supporting Information

Buried interfaces in organic photocathodes for H_2 evolution: Fermi level pinning and recombination

Eui Hyun Suh[1], Michel De Keersmaecker[1], Ratul Mitra Thakur[1], Bo Dong[2], Tianquan Lian[2], Neal R. Armstrong[3], and Erin L. Ratcliff[1,4,]*

[1]School of Materials Science and Engineering, Georgia Institute of Technology, Atlanta, GA 30332, *United States*

[2]Department of Chemistry, University of Pennsylvania, Philadelphia, PA 19104, *United States*

[3]Department of Chemistry and Biochemistry, University of Arizona, Tucson, AZ 85721, *United States*

[4]School of Chemistry and Biochemistry, Georgia Institute of Technology, Atlanta, GA 30332, *United States*

Corresponding Author

*Email: eratcliff8@gatech.edu.

Table of Contents

1 Experimental section 3

1.1 Materials 3

1.2 Surface treatments for ITO, FTO, and ITO/NiO$_x$ substrates 4

1.3 Buk heterojunction deposition 4

1.4 Photoelectrochemical Pt deposition 4

1.5 Photoelectrochemical device characterization 5

1.6 Characterization procedures 6

1.6.1 Cyclic voltammetry (CV) 6
1.6.2 Ultraviolet photoelectron spectroscopy (UPS) 6
1.6.3 X-ray photoelectron spectroscopy (XPS) 6
1.6.4 Contact angle measurements 7
1.6.5 UV-vis-NIR spectroscopy 7
1.6.6 Transient Absorption experiment 7
1.6.7 OCP measurement 7
1.6.8 H_2 production measurement 8
1.6.9 Electrochemical Quartz Crystal Microbalance 8

2 Supplementary Note 1: Alternative blended heterojunctions and contacts 9

3 Supplementary Note 2: Energetics of PTB7-Th, P(NDI2OD-T2), and Contacts 11

4 Supplementary Note 3: Characterizations of BHJs and Photocathodes 12

5 Supplementary Note 4: The issue of detachments of H_2 gas bubbles. 19

6 Supplemental Note 5: XPS analysis using C 1s, P 2p, F 1s, Sn 3d and In 3d core level spectra20

7 Supplementary Note 6: Evidence of electrolyte penetration in photocathodes 24

8 Supplementary Note 7: Time-dependent carrier recombination 29

9 Supplemental Note 8: FcOH/FcOH$^+$ redox chemistry on hole-only devices in the dark 34

10 Supporting References 39

1 Experimental section

1.1 Materials

Butylphosphonic acid, 2,3,4,5,6-penfluorophenylphosphonic acid (97%), potassium tetrachloroplatinate(II) (K_2PtCl_4, 98%), ferrocenemethanol (97%), benzoquinone (≥ 98%), and 1-choronaphthalene (1-CN) were purchased from Sigma-Aldrich. [2-(9*H*-Cabazol-9-yl)ethyl]phosphonic acid (2PACz; > 98.0%) was purchased from TCI. PTB7-Th (M0261A4; M_w = 125,205 g mol^{-1} and PDI = 2.63) and P(NDI2OD-T2) (M1201A2; M_w =125,509 g mol^{-1} and PDI = 2.3) were purchased from Ossila. Poly[(2,6-(4,8-bis(5-(2-ethylhexyl)-4-fluorothiophen-2-yl)-benzo[1,2-*b*:4,5-*b*']dithiophene))-*alt*-(5,5-(1',3'-di-2-thienyl-5',7'-bis(2-ethylhexyl)benzo [1',2'-*c*:4',5'-*c*']dithiophene-4,8-dione))] (PM6) was purchased from 1-Material (YY20182CH100; M_w = 100,000 g mol^{-1} and PDI = 2.3). 2,2'-((2Z,2'Z)-((12,13-bis(2-ethylhexyl)-3,9-diundecyl-12,13-dihydro-[1,2,5]thiadiazolo[3,4-*e*]thieno[2",3'':4',5']thieno[2',3':4,5]pyrrolo [3,2-*g*]thieno[2',3':4,5]thieno[3,2-*b*]indole-2,10-diyl)bis(methanylylidene))bis(5,6-difluoro-3-oxo -2,3-dihydro-1*H*-indene-2,1-diylidene))dimalononitrile (Y6) was purchased from Brilliant Matters (BTPF03, > 99%). Poly[[5,6-difluoro-2-(2-hexyldecyl)-2*H*-benzotriazole-4,7-diyl]-2,5-thiophenediyl[4,8-bis[5-(2-ethylhexyl)-2-thienyl]benzo[1,2-*b*:4,5-*b*']dithiophene-2,6-diyl]-2,5-thiophenediyl] (J52) was purchased from Lumtec (S9469-19, M_w > 10,000 g mol^{-1}). Hydroiodic acid (57%) was purchased from Emplura. Sodium sulfate (Na_2SO_4, ACS 99.0%), chlorobezene (99.5% extra dry), and 2,2,2-trifluoroethanol (99.8 extra pure) were purchased from Thermo Scientific. All chemicals were used without further purification.

1.2 Surface treatments for ITO, FTO, and ITO/NiO_x substrates

ITO and FTO (1.5 × 1.5 cm^2) were successively bath-sonicated in DI water with 1 vol% Triton X-100, acetone, and isopropyl alcohol for 20 min, respectively. For HI treatment, cleaned ITO was dipped into HI solution for 8 s and washed by DI water. For UV-ozone treatment, cleaned ITO was UV-ozone treated for 20 min. For Butyl-PA and PhF_5-PA treatments, Butyl-PA and PhF_5-PA were dissolved in trifluoroethanol (2 mM) at 80 °C for 2 h, respectively. The solutions were spin-coated on UV-ozone-treated ITO at 3000 rpm for 30 s. The PA-SAM films were annealed at 120°C for 5 min. After annealing, the films were washed by dynamic spin coating at 6000 rpm for 40 s. Pure trifluoroethanol (50 µL) was dropped on the films two times at 15 s and 30 s, respectively. NiO_x solution was prepared and deposited on ITO as reported in our previous work.[1]

1.3 Buk heterojunction deposition

PTB7-Th and P(NDI2OD-T2) were dissolved in chlorobenzene (PTB7-Th:P(NDI2OD-T2) = 2:1 wt/wt; 8 mg mL^{-1}) at 50°C and the solution was stirred at 800 rpm for overnight. The blend solution was spin-coated on pre-treated ITO at 2000 rpm for 60 s. After spin coating, the films were annealed at 100 °C for 10 min. For other BHJ layers, PM6 and Y6 were dissolved in chloroform (PM6:Y6 = 1:1.2 wt/wt; 10 mg mL^{-1}) and J52 and P(NDI2OD-T2) were dissolved in chlorobenzene (J52:P(NDI2OD-T2) = 2:1 wt/wt; 8 mg mL^{-1}), respectively. 5 vol/vol % of 1-CN was added to PM6:Y6 solution. The PM:Y6 and J52:P(NDI2OD-T2) solutions were spin-coated on pre-treated ITO at 3000 rpm for 30 s and 2000 rpm for 60 s, respectively. All films were annealed at 100°C for 10 min.

1.4 Photoelectrochemical Pt deposition

Pt nanoparticles were deposited by photoelectrochemical reduction in a three-electrode system. ITO/BHJ and Ag/AgCl/sat. KCl/0.5 M Na_2SO_4 were used as working and reference electrodes,

respectively. IrO_x-Ta_2O_x-deposited Ti plate was used as a counter electrode to suppress metal dissolution from counter electrodes and re-deposition to working electrodes. 0.5 mM K_2PtCl_4 and 0.5 M Na_2SO_4 were simultaneously dissolved in DI water to use as an electrolyte. Before Pt deposition, the electrolyte was Ar-sparged for at least 30 min. White LED (MWWHL3, Thorlabs) was used as a light source. The LED was 19 mm away from the working electrode. Estimated light intensity is 100 mW cm^{-2}, verified with a calibrated Si photodiode. Constant potentials were applied to the working electrodes with an active area of 0.672 cm^2 (0.1 V for SC and HI samples, 0.2 V for UV-ozone sample, and 0.3 V for Butyl-PA and PhF_5-PA samples, respectively) using a CH Instruments 920D bipotentiostat. Estimated Pt loading is 15 mC cm^{-2} which was calculated by integrated area of *J*-*t* curves during chronoamperometry.

1.5 Photoelectrochemical device characterization

Hydrogen evolution reaction (HER) performances were measured in a three-electrodes system. ITO/BHJ/Pt, Ag/AgCl/sat. KCl/0.1 M H_2SO_4, IrO_x-Ta_2O_x-deposited Ti plate were used as working, reference, and counter electrodes, respectively. 0.1 M H_2SO_4 in DI water was used as an electrolyte. The electrolyte was Ar-sparged for at least 30 min and refilled before every LSV scan. A solar simulator with AM1.5G-filtered Xe lamp (HAL-320, Asahi spectra) and a light chopper (2 Hz) was used as a light source. LSV scan was performed at scan rate of 20 mV s^{-1} with an active area of 0.672 cm^2. ABPE was calculated as following equation: $J \cdot V_{RHE}/100$ mW cm^{-2} $\times 100$ (%). V_{onset} was extracted by linear fitting of *J*-*V* curves near half maximum current densities. Other characterization details are summarized below in the Section 1.6.

1.6 Characterization procedures

1.6.1 Cyclic voltammetry (CV)

CV was performed in a three-electrodes system. ITO/PTB7-Th (and ITO/P(NDI2OD-T2)), Ag/AgCl/sat. KCl, and Pt foil were used as working, reference, and counter electrodes, respectively. 0.1 M H_2SO_4 solution in DI water was used as an electrolyte. For CV scans with redox couples (ferrocenemethanol (FcOH) and benzoquinone (BQ)), ITO (and ITO/BHJ), Ag/AgCl/sat. KCl, and IrO_x-Ta_2O_x-coated Ti plate were used as working, reference and counter electrodes, respectively. 0.1 M H_2SO_4+1 mM FcOH (or 1mM BQ) solutions in DI water were used as an electrolyte.

1.6.2 Ultraviolet photoelectron spectroscopy (UPS)

UPS (He I excitation at 21.22 eV, pass energy of 5 eV) spectra were acquired at a photoelectron takeoff angle of 90° (normal to the surface) using a Kratos Axis Ultra PES system (Kratos Analytical, USA) in ultra-high vacuum with a base pressure around 2×10^{-9} Torr. The He discharge lamp (SPECS UVS 10/35, 25 mA) was operated in a chamber pressure of 1×10^{-7} Torr using ultra high purity (UHP) He which runs through a liquid nitrogen-cooled trap to remove impurities. All samples were fixed using carbon tape and grounded to stainless steel stubs. The Fermi energy of the instrument was calibrated with Au film. The samples were biased at −10.0 V to enhance the photoelectron yield at the low kinetic energy edge (LKE).

1.6.3 X-ray photoelectron spectroscopy (XPS)

XPS (monochromatic $Al_{K\alpha}$ excitation at 1486.3 eV, 10 mA, 15 kV, and pass energy of 20 eV) spectra were acquired at a photoelectron takeoff angle of 90° (normal to the surface) using a Kratos Axis Ultra PES system (Kratos Analytical, USA) in ultra-high vacuum with a base pressure around 2×10^{-9} Torr. All samples were fixed using carbon tape and grounded to stainless steel stubs. The raw XPS data were corrected by Shirley background. Charge correction was performed with adventitious C 1s and C-C peaks at a binding energy of 284.8 eV. The XPS spectra were fit with Gaussian 70% and Lorentzian 30% functions.

1.6.4 Contact angle measurements

Water contact angle measurement was performed with a contact angle goniometer (Rame-Hart Model 250).

1.6.5 UV-vis-NIR spectroscopy

UV-vis-NIR spectroscopy was performed with an Agilent 8453 spectrophotometer. BHJ layer thickness was measured by a surface profiler (KLA-Tencor P15 profilometer).

1.6.6 Transient Absorption experiment

The Astrella ultrafast Ti:sapphire amplifier (Coherent) was used to generate the fundamental laser beam (800 nm nominal center wavelength, 5 W power output, 35 fs pulse width, 1 kHz repetition rate). 2.35 W of the fundamental beam was directed into a tunable visible optical parametric amplifier (OPerA, Coherent) to generate 650-nm excitation light. Specifically, the 650-nm light was produced by second harmonic generation of the signal beam through a BBO crystal inside the OPA. The 800-nm fundamental and 650-nm pump laser were aligned into a Helios Fire transient absorption spectrometer (Ultrafast Systems) for TA experiments. The 800-nm fundamental, after traveling through a motorized delay stage, was focused onto a CaF_2 window to produce white light continuum (WLC), which served as the probe beam. The pump beams, chopped at a rate of 500 Hz, were aligned to overlap with the probe beams at the sample stage. Signal detection was performed using linear array detectors with InGaAs sensors. Since every other pump beam was blocked from the sample by a chopper, two consecutive probe pulses were used to calculate the ΔA spectrum. All the TA experiments were carried out in ambient air.

1.6.7 OCP measurement

For transient open circuit potential (OCP) measurement, 95/5 N_2/H_2 mixed gas sparged into the electrolyte during measurement to make quasi-equilibrium environment for HER (**Figure S15a**). All photocathodes were LSV scanned five times to saturate H_2 gas on them. EIS was measured in a three-electrodes system. ITO/BHJ/Pt, Ag/AgCl/sat. KCl/0.1 M H_2SO_4, IrO_x-Ta_2O_x-deposited Ti plate were used as working, reference, and counter electrodes, respectively. 0.1 M H_2SO_4 in DI water was used as an electrolyte. The electrolyte was Ar-sparged for at least 30 min and refilled before every LSV scan. Frequency was swept from 1 MHz to 0.1 Hz applying 0.25 V_{RHE} under 1 sun illumination.

1.6.8 H_2 production measurement

H_2 gas was quantified by gas chromatography (SRI GC 8610C). H-cell was built to capture H_2 gas from the photocathodes. ITO/BHJ/Pt, IrO_x-Ta_2O_x-coated Ti plate, and Ag/AgCl/sat. KCl were used as a working, counter, and reference electrodes, respectively. Nafion (SOLVAY AQUIVION^R) was used as a membrane. 0.1 M H_2SO_4 in DI water was used as an electrolyte, which Ar-sparged for 30 min before measurement. The electrolyte additionally Ar-sparged in the H-cell for 5 min to remove air in the H-cell. The gas of 50 µL was extracted from the H-cell and injected into the GC using a gas-tight syringe to measure the concentration of H_2 gas.

1.6.9 Electrochemical Quartz Crystal Microbalance

EQCM measurements were performed with a Gamry interface 1010E potentiostat coupled to a Gamry eQCM-I Mini. A BHJ film was spin-coated onto a 5MHz Cr/Au EQCM substrate in a manner consistent with BHJ application on ITO substrates. The film was slowly heated at a ramp rate of 2 °C min^{-1} and annealed at 50 °C for 1 h to minimize thermal damage of the EQCM substrate. The EQCM substrate was purchased from Quartz Pro. The BHJ film was further vacuum-dried for overnight to remove residual water. A Pt wire and Ag/AgCl/sat. KCl were used as a counter and reference electrodes, respectively. 0.1 M H_2SO_4 in DI water was used as an electrolyte, which Ar-sparged for 30 min before measurement. Cyclic voltammetry was performed from 0.96 V to 0.06 V_{RHE} for 10 cycles at a scan rate of 20 mV s^{-1} under dark condition. Prior to CV scans, the samples were kept in the electrolyte for 600 s to allow the frequency of the EQCM substrate to stabilize. Mass change (Δm) was calculated using Sauerbrey equation: $\Delta m = -C\times\Delta f$, where C is the mass sensitivity constant (17.7 ng cm^{-2} Hz^{-1}) and Δf is the frequency change of the EQCM substrate.

2 Supplementary Note 1: Alternative blended heterojunctions and contacts

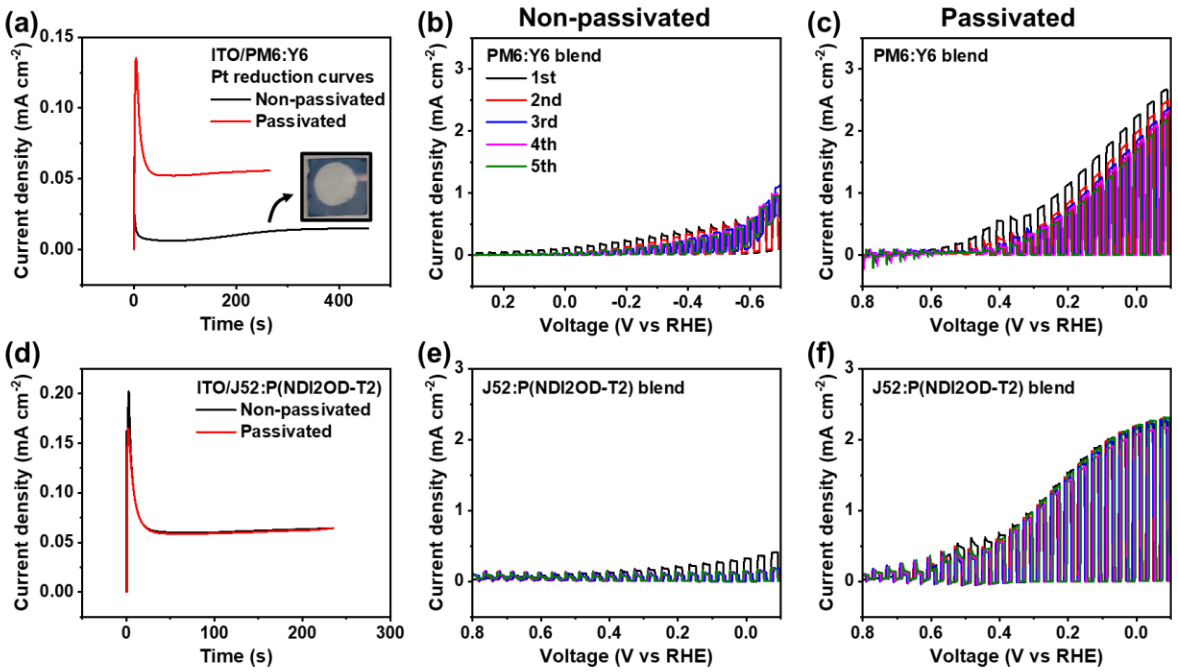

Figure S1. CA curves for Pt reduction reaction on photocathodes (ITO/BHJ) with (a) PM6:Y6 and (d) J52:P(NDI2OD-T2) BHJ layers at 0.2 VAg/AgCl. J-V curves of (b,e) non-passivated (UV-ozone) and (c,f) passivated (Butyl-PA) photocathodes (ITO/BHJ/Pt) with (b,c) PM6:Y6 and (e,f) J52:P(NDI2OD-T2) blends. All photocathodes were fabricated in ambient air. Inset in (a): a photograph of one of UV-ozone photocathodes with PM6:Y6 blend after Pt deposition.

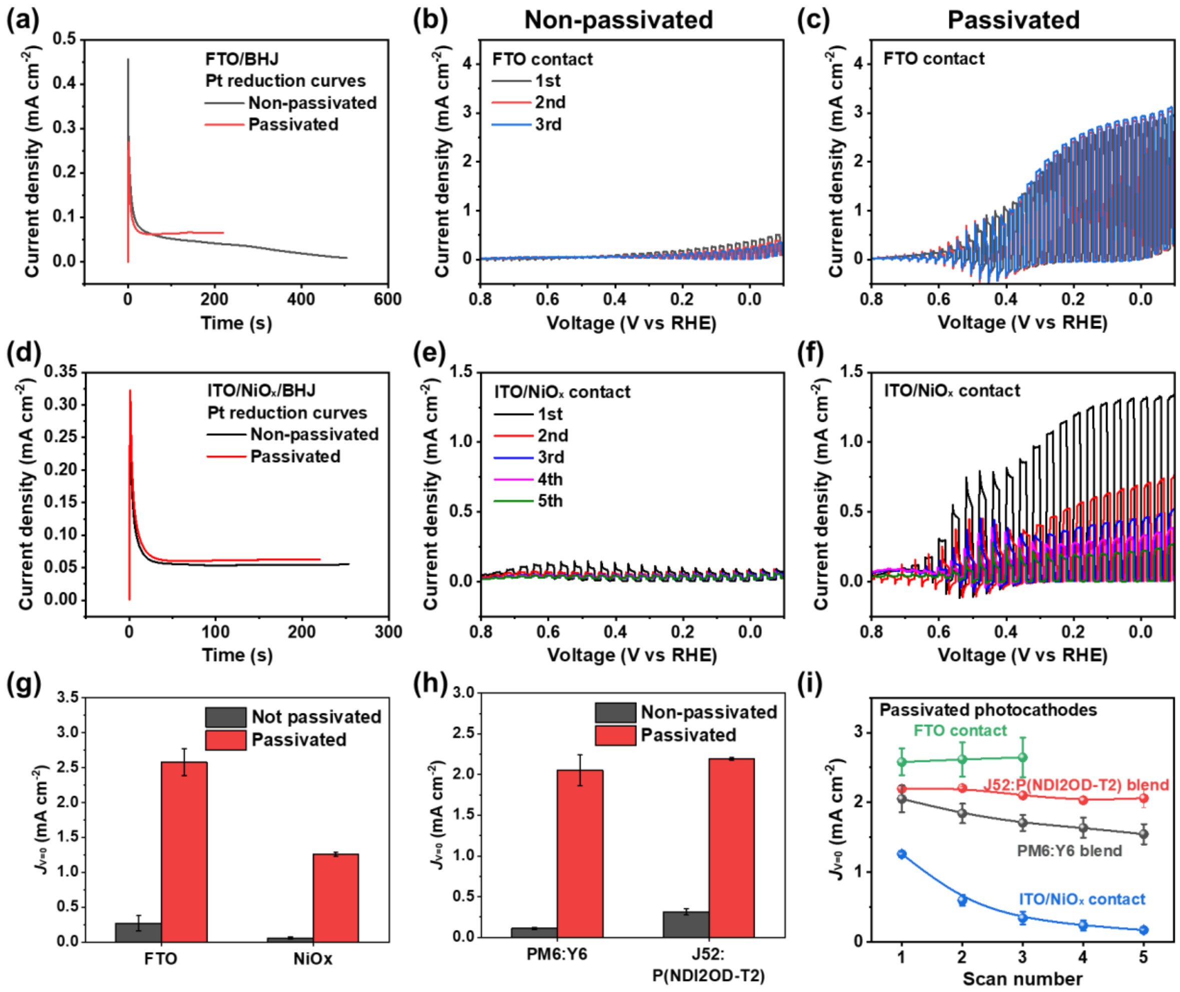

Figure S2. CA curves for Pt reduction reaction on (a) FTO and (d) NiO_x photocathodes (FTO/BHJ and ITO/NiO_x/BHJ, respectively) at 0.3 VAg/AgCl. J-V curves of (b,c) FTO and (d,e) NiO_x photocathodes (b,e) without and (c,f) with PA-SAM passivation, respectively (FTO/BHJ/Pt and ITO/NiO_x/BHJ/Pt). 2PACz and Butyl-PA were used for FTO and ITO/NiO_x contacts, respectively. Summary of $J_{V=0}$ of photocathodes with different (g) oxide contacts and (h) BHJ layers. (i) $J_{V=0}$ plot as a function of LSV scan number. $J_{V=0}$ of several photocathodes with different (g) oxide contacts and (h) BHJ layers. (i) $J_{V=0}$ plot of PA-SAM-passivated photocathodes as a function of scan number. All photocathodes were fabricated in ambient air except for FTO photocathodes. FTO photocathodes were fabricated in a N_2-filled glove box.

3 Supplementary Note 2: Energetics of PTB7-Th, P(NDI2OD-T2), and Contacts

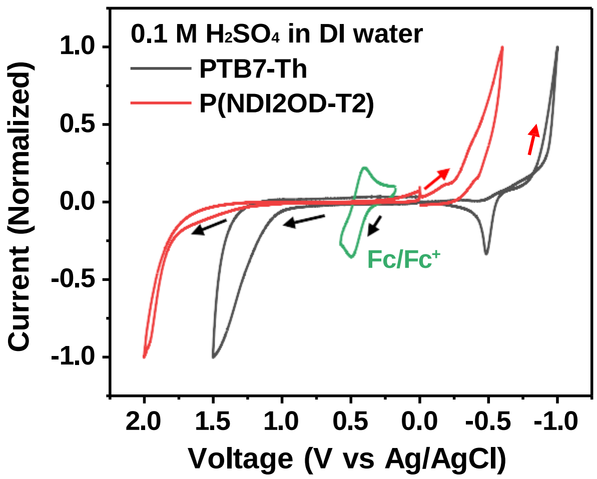

Figure S3. Cyclic voltammograms (CVs) of PTB7-Th and P(NDI2OD-T2) films in aqueous 0.1 M H_2SO_4 electrolyte and ferrocene in 0.1 M $TBAPF_6$/acetonitrile, respectively, at a scan rate of 50 mV s^{-1}. The Fc/Fc^+ couple was used to standardize the Ag/AgCl reference electrode and provide comparison to the vacuum scale ($E_{1/2, Fc/Fc+}$ = –4.8 eV).[2]

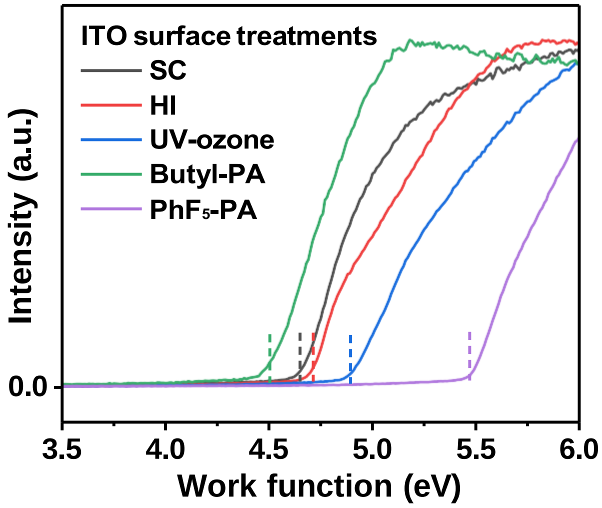

Figure S4. Ultraviolet photoelectron spectroscopy (UPS) spectra of chemically altered ITO surfaces in secondary electron cutoff region.

4 Supplementary Note 3: Characterizations of BHJs and Photocathodes

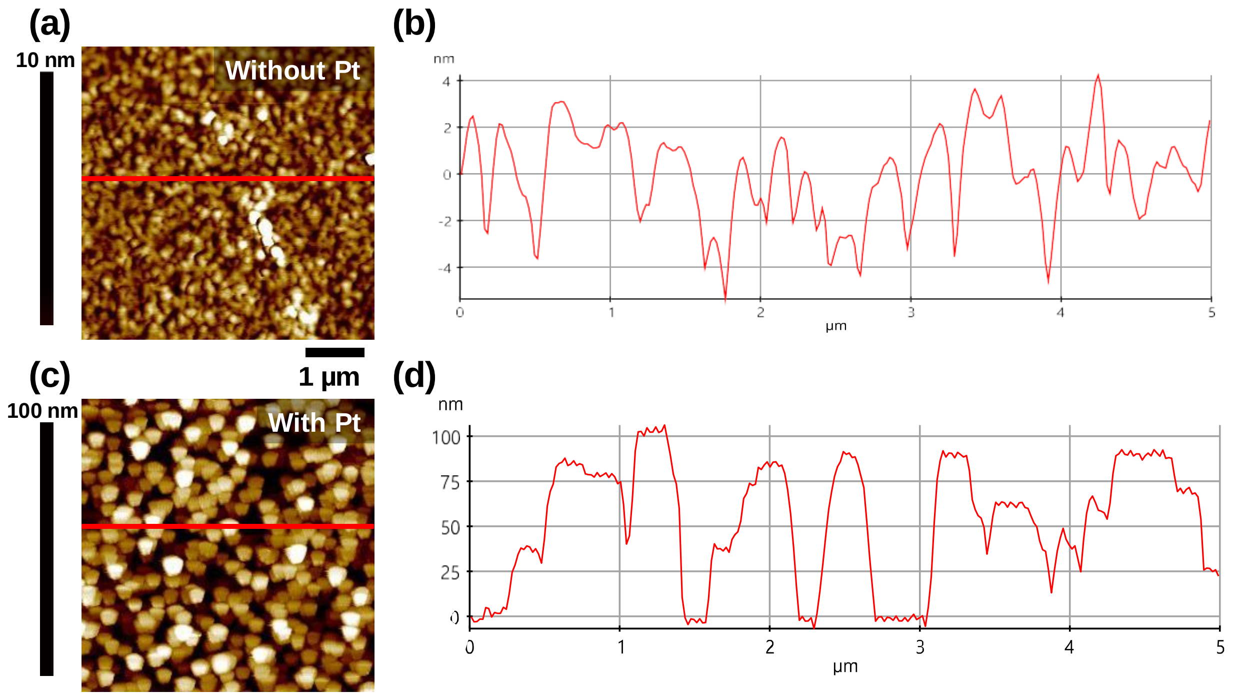

Figure S5. AFM topographic images of (a) ITO/BHJ and (c) ITO/BHJ/Pt. Line-cut profiles of (b) ITO/BHJ and (d) ITO/BHJ/Pt (red lines in (a) and (b)).

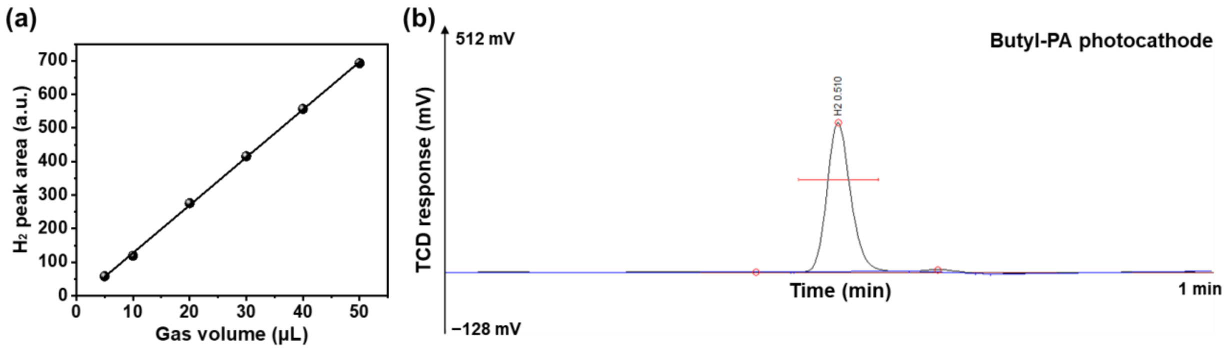

Figure S6. (a) GC calibration curve using N_2/H_2 mixed gas for H_2 quantification. (b) GC chromatogram of the gas components in the H-cell with Butyl-PA photocathode.

Discussion: To determine the H_2 evolution rates and Faradaic efficiencies of the photocathodes, a calibration curve was established using N_2/H_2 mixed gas (Figure S6a). The calculations were performed according to the following equations:

$$\text{Faradaic efficiency (\%)} = \frac{V_{\text{measured}}}{V_{\text{estimated}}} \times 100$$

where V_{measured} is the volume of H_2 directly quantified via GC and $V_{\text{estimated}}$ is the theoretical volume of H_2 calculated from the time integrated photocurrent charge, assuming a 100% Faradaic efficiency.

$$V_{\text{measured}} = V_{\text{GC}} \cdot C_{\text{mixed gas}} \cdot \frac{V_{\text{empty}}}{V_{\text{injected}}}$$

where V_{GC} is the H_2 volume determined from the GC peak area (Figure S6b) using the linear fitting from the calibration curve (Figure S6a), $C_{\text{mixed gas}}$ is the concentration of H_2 in the mixed gas, V_{empty} is the empty volume of the H-cell, and V_{injected} is the specific gas volume injected into the GC.

$$V_{\text{estimated}} = \frac{Q}{2} \cdot \frac{1}{F} \cdot \frac{RT}{P}$$

where Q is the time-integrated charge passed during chronoamperometry scan, F is the Faraday constant, R is the ideal gas constant, T is the absolute temperature, and P is the atmospheric pressure. The factor of 2 in the denominator accounts for the two electrons required to reduce two protons to a single H_2 molecule. As a control experiment, a near 100% Faradaic efficiency was confirmed when a Pt foil was used as the working electrode to generate H_2 gas electrochemically.

Table S1. Summary of reported H_2 evolution rates of soft photoelectrodes and photocatalysts.

	Materials	H_2 evolution rates (μmol h^{-1} cm^{-2})	Reference
Photoelectrodes	ITO/Butyl-PA/PTB7-Th:P(NDI2OD-T2)/Pt	20.25 ± 0.20	This work
	ITO/PhF_5-PA/PTB7-Th:P(NDI2OD-T2)/Pt	12.20 ± 0.02	This work
	FTO/MoO_3/PTB7-Th:PDI-V/RuO_2	149[a]	J. Am. Chem. Soc. 2020, 142, 7795
	ITO/x-PEDOT:PSS/P3HT:PCBM/TiO_x/Pt	1.5	J. Phys. Chem. C, 2015, 119, 6488
	FTO/CuO_x/PTB7-Th:PCBM/TiO_x/Pt	35	Chem. Mater. 2019, 31, 1928
	FTO/2PACz/PBDTTTPD:PNDIHDT/RuO_2	95.83	Adv. Energy. Mater. 2022, 12, 2202363
	FTO/Polythiophene	1.8	Cell Rep. Phys. Sci. 2021, 2, 100306
Photocatalysts	PTB7-Th:EH-IDTBR (Pt)	28.52	Nat. Mater. 2020, 19, 559
	PM6:PCBM (Pt)	16.7	Nat. Energy. 2022, 7, 340
	PDI	9.10	Nat. Commun. 2024, 15, 5047
	PM6:ITCC-M:IDMIC-4F (Pt)	17	Adv. Funct. Mater. 2022, 33, 2209643
	T1-75 (Pt)	2	Adv. Mater. 2024, 36, 2300037
	gIDTBT:oIDTBR (Pt)	4.20	Adv. Mater. 2022, 34, 2105007
	P10 (trace Pd)	15.33	J. Am. Chem. Soc. 2020, 142, 14574
	PTB-eC9-B4F (Pt)	1.05	Dyes. Pigm. 2024, 224, 111982
	PM6:Y6CO (Pt)	2.79	Angew. Chem. Int. Ed. 2023, 62, e202217989

[a] Estimated by current densities

Discussion:

H_2 evolution rates of several soft photoelectrodes and photocatalysts are compared in **Table S1**. Area-normalized values are used to compare the two different water splitting system approaches (i.e., electrodes and colloidal suspensions), although direct comparisons between rates should be done with exercised caution. Photoelectrodes characterized as half-cells generally apply 0 V_{RHE}, which is different than device operation conditions for unassisted water splitting. Soft

photoelectrodes generally do not have enough photovoltages (< 1.0 V) to split water (1.5 V) without hole scavengers or external bias. Photocatalyst literature predominantly uses hole scavengers (e.g., ascorbic acid) to evolve H_2 gas. In general, more factors need to be considered, such as electrolyte pH, the presence of noble metal catalysts, polymer nanoparticle concentrations to evaluate device performances.

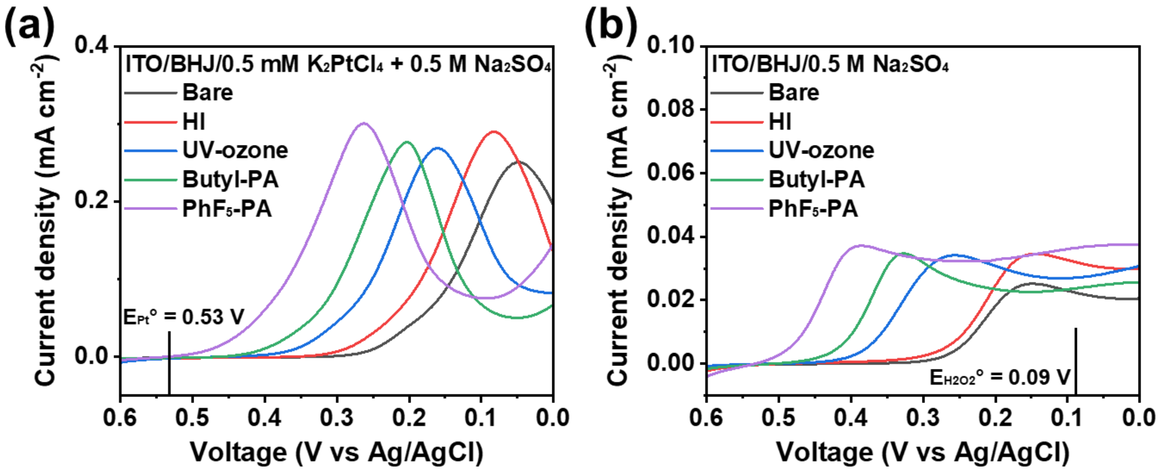

Figure S7. *J-V* curves of photocathodes (ITO/BHJ) LSV scanned in aqueous 0.5 M Na_2SO_4 electrolyte (a) with and (b) without Pt precursor (0.5 mM K_2PtCl_4) at a scan rate of 50 mV s^{-1}.

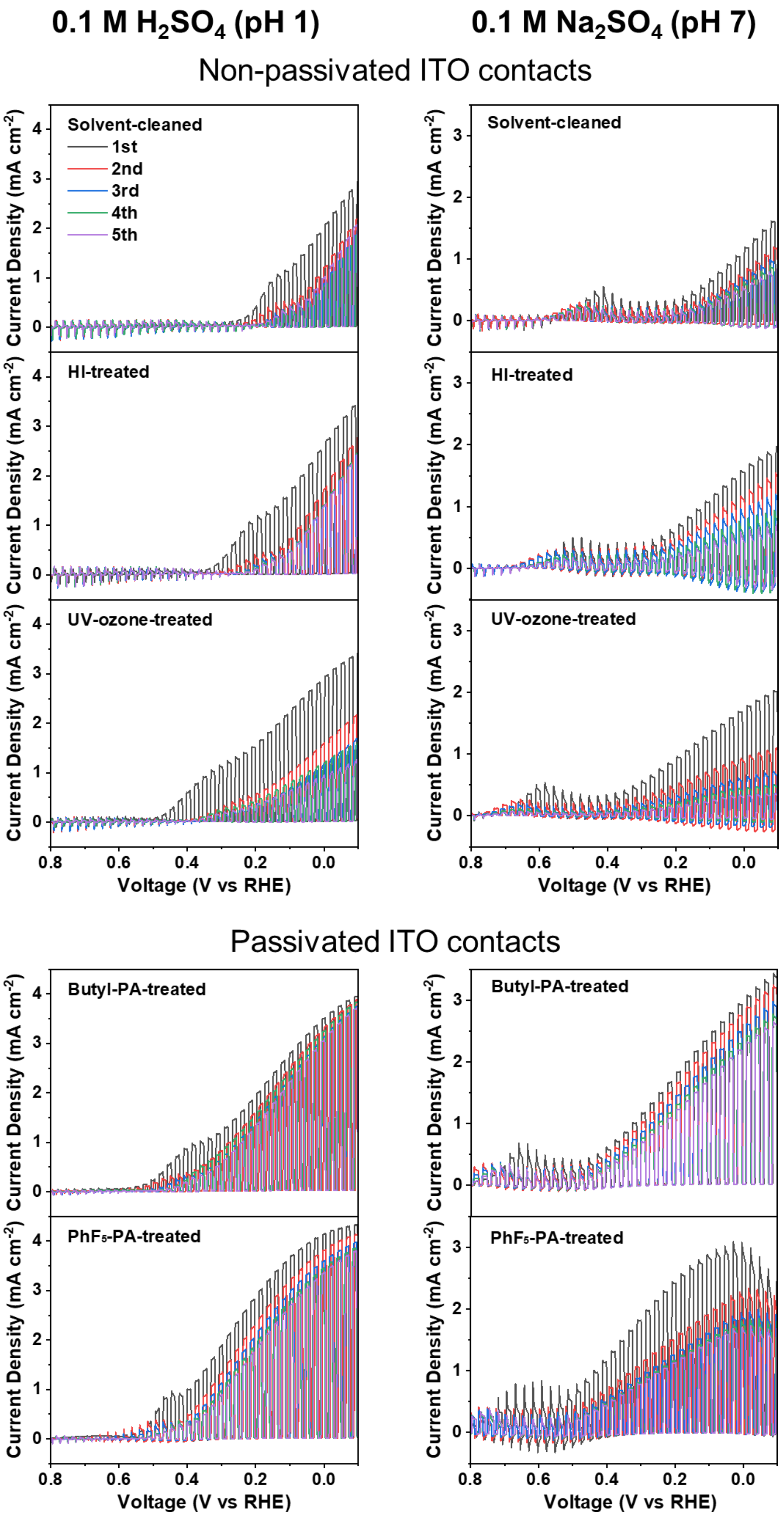

Figure S8. Sequential J-V curves of photocathodes performed in aqueous 0.1 M H_2SO_4 (left column) and 0.1 M Na_2SO_4 (right column) electrolytes comprised of non-passivated ITO contacts (a,b) SC, (c,d) HI, (e,f) UV-ozone, and ITO contacts passivated with (g,h) Butyl-PA and (i,j) PhF_5-PA.

Table S2. HER performances of photocathodes with surface-treated ITO substrates in acidic pH (aqueous 0.1 M H_2SO_4); standard deviations of 3 devices is provided as ± the value.

		$J_{V=0}$ (mA cm^{-2})	V_{onset} (V_{RHE})	ABPE (%)
SC	1st	1.97 ± 0.07	0.22 ± 0.01	0.14 ± 0.01
	2nd	1.21 ± 0.01	0.12 ± 0.01	0.06 ± 0.01
	3rd	0.93 ± 0.07	0.10 ± 0.01	0.04 ± 0.01
	4th	0.94 ± 0.12	0.09 ± 0.02	0.04 ± 0.01
	5th	1.07 ± 0.09	0.10 ± 0.02	0.04 ± 0.01
HI	1st	2.85 ± 0.32	0.33 ± 0.04	0.27 ± 0.04
	2nd	1.94 ± 0.28	0.20 ± 0.03	0.10 ± 0.02
	3rd	1.55 ± 0.21	0.16 ± 0.02	0.07 ± 0.01
	4th	1.44 ± 0.09	0.14 ± 0.01	0.07 ± 0.02
	5th	1.43 ± 0.05	0.14 ± 0.01	0.07 ± 0.02
UV-ozone	1st	2.88 ± 0.17	0.41 ± 0.04	0.30 ± 0.02
	2nd	1.74 ± 0.12	0.26 ± 0.03	0.12 ± 0.01
	3rd	1.40 ± 0.16	0.24 ± 0.03	0.15 ± 0.02
	4th	1.19 ± 0.05	0.26 ± 0.02	0.16 ± 0.01
	5th	1.02 ± 0.16	0.26 ± 0.02	0.16 ± 0.01
Butyl-PA	1st	3.56 ± 0.34	0.42 ± 0.02	0.39 ± 0.03
	2nd	3.35 ± 0.32	0.38 ± 0.02	0.33 ± 0.01
	3rd	3.20 ± 0.31	0.36 ± 0.02	0.30 ± 0.01
	4th	3.15 ± 0.26	0.36 ± 0.02	0.29 ± 0.01
	5th	3.11 ± 0.23	0.35 ± 0.02	0.28 ± 0.01
PhF_5-PA	1st	4.07 ± 0.24	0.54 ± 0.03	0.63 ± 0.03
	2nd	3.68 ± 0.09	0.47 ± 0.03	0.51 ± 0.03
	3rd	3.67 ± 0.12	0.45 ± 0.03	0.47 ± 0.04
	4th	3.54 ± 0.07	0.44 ± 0.03	0.44 ± 0.05
	5th	3.44 ± 0.13	0.43 ± 0.03	0.41 ± 0.04

Table S3. HER performances of photocathodes with surface-treated ITOs in neutral pH (aqueous 0.1 M Na_2SO_4).

		$J_{V=0}$ (mA cm^{-2})	V_{onset} (V_{RHE})	ABPE (%)
**SC**	**1st**	1.13 ± 0.11	0.23 ± 0.01	0.07 ± 0.008
	**2nd**	0.84 ± 0.07	0.19 ± 0.005	0.05 ± 0.003
	**3rd**	0.75 ± 0.05	0.18 ± 0.003	0.04 ± 0.003
	**4th**	0.66 ± 0.08	0.18 ± 0.001	0.04 ± 0.003
	**5th**	0.60 ± 0.08	0.18 ± 0.006	0.04 ± 0.003
**HI**	**1st**	1.66 ± 0.10	0.36 ± 0.02	0.15 ± 0.01
	**2nd**	1.09 ± 0.24	0.34 ± 0.04	0.10 ± 0.01
	**3rd**	0.76 ± 0.21	0.29 ± 0.04	0.07 ± 0.02
	**4th**	0.56 ± 0.22	0.29 ± 0.05	0.05 ± 0.02
	**5th**	0.39 ± 0.15	0.29 ± 0.06	0.04 ± 0.01
**UV-ozone**	**1st**	1.60 ± 0.21	0.46 ± 0.03	0.20 ± 0.02
	**2nd**	0.57 ± 0.27	0.42 ± 0.02	0.07 ± 0.03
	**3rd**	0.31 ± 0.18	0.40 ± 0.03	0.04 ± 0.02
	**4th**	0.23 ± 0.13	0.41 ± 0.01	0.03 ± 0.01
	**5th**	0.16 ± 0.10	0.38 ± 0.01	0.02 ± 0.01
**Butyl-PA**	**1st**	2.84 ± 0.09	0.50 ± 0.02	0.37 ± 0.003
	**2nd**	2.64 ± 0.11	0.48 ± 0.01	0.32 ± 0.01
	**3rd**	2.42 ± 0.07	0.46 ± 0.003	0.29 ± 0.007
	**4th**	2.23 ± 0.08	0.45 ± 0.001	0.25 ± 0.008
	**5th**	2.14 ± 0.05	0.43 ± 0.001	0.23 ± 0.009
**PhF_5-PA**	**1st**	2.59 ± 0.54	0.59 ± 0.007	0.53 ± 0.06
	**2nd**	1.86 ± 0.51	0.56 ± 0.004	0.31 ± 0.06
	**3rd**	1.62 ± 0.40	0.55 ± 0.006	0.26 ± 0.04
	**4th**	1.54 ± 0.38	0.54 ± 0.004	0.24 ± 0.04
	**5th**	1.46 ± 0.35	0.54 ± 0.003	0.24 ± 0.04

5 Supplementary Note 4: The issue of detachments of H_2 gas bubbles.

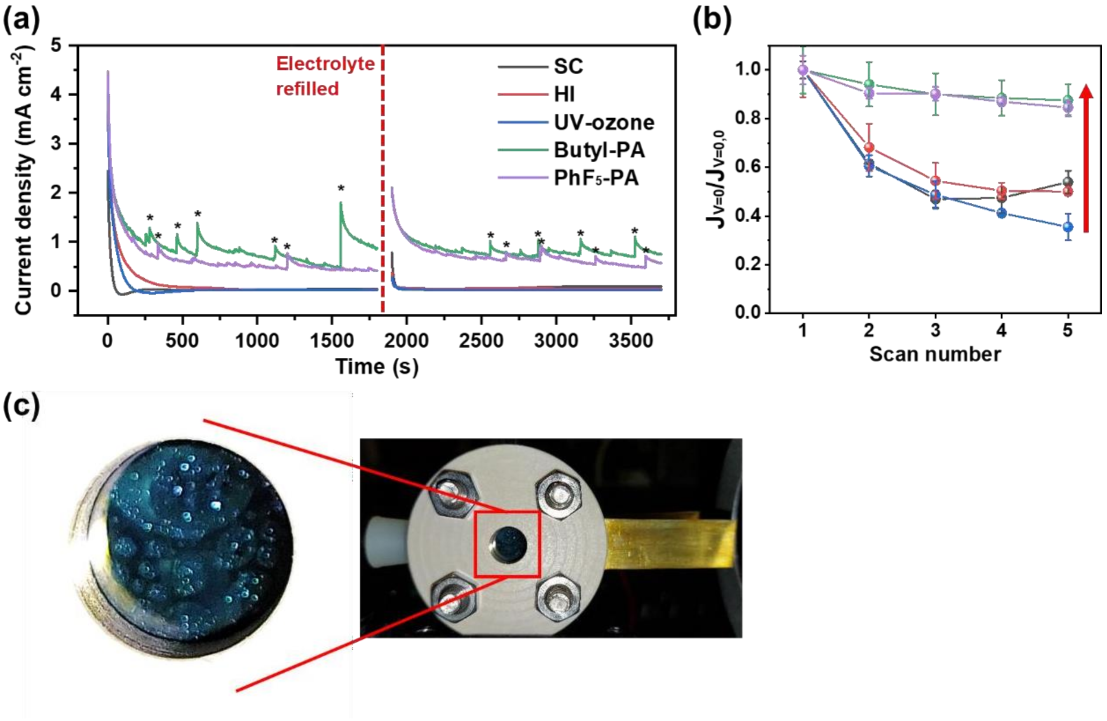

Figure S9. The stability of the photocathode is measured in (a) chronoamperometry scans and (b) $J_{V=0}$ plots as a function of LSV scan number in aqueous 0.1 M H_2SO_4 electrolyte recorded under 1 sun illumination. Bubble detachment in (a) produce current spikes (*) due to the increased surface area. (c) Picture of bubbles stuck on the surface of the photoactive layer under operation.

Figure S9c demonstrates an inherent problem with hydrophobic soft photocathodes in that the bubbles do not readily desorb from the active layer/catalytic sits, but do so somewhat sporadically, yielding the current density spikes versus time emphasized in Figure S9b.

6 Supplemental Note 5: XPS analysis using C 1s, P 2p, F 1s, Sn 3d and In 3d core level spectra

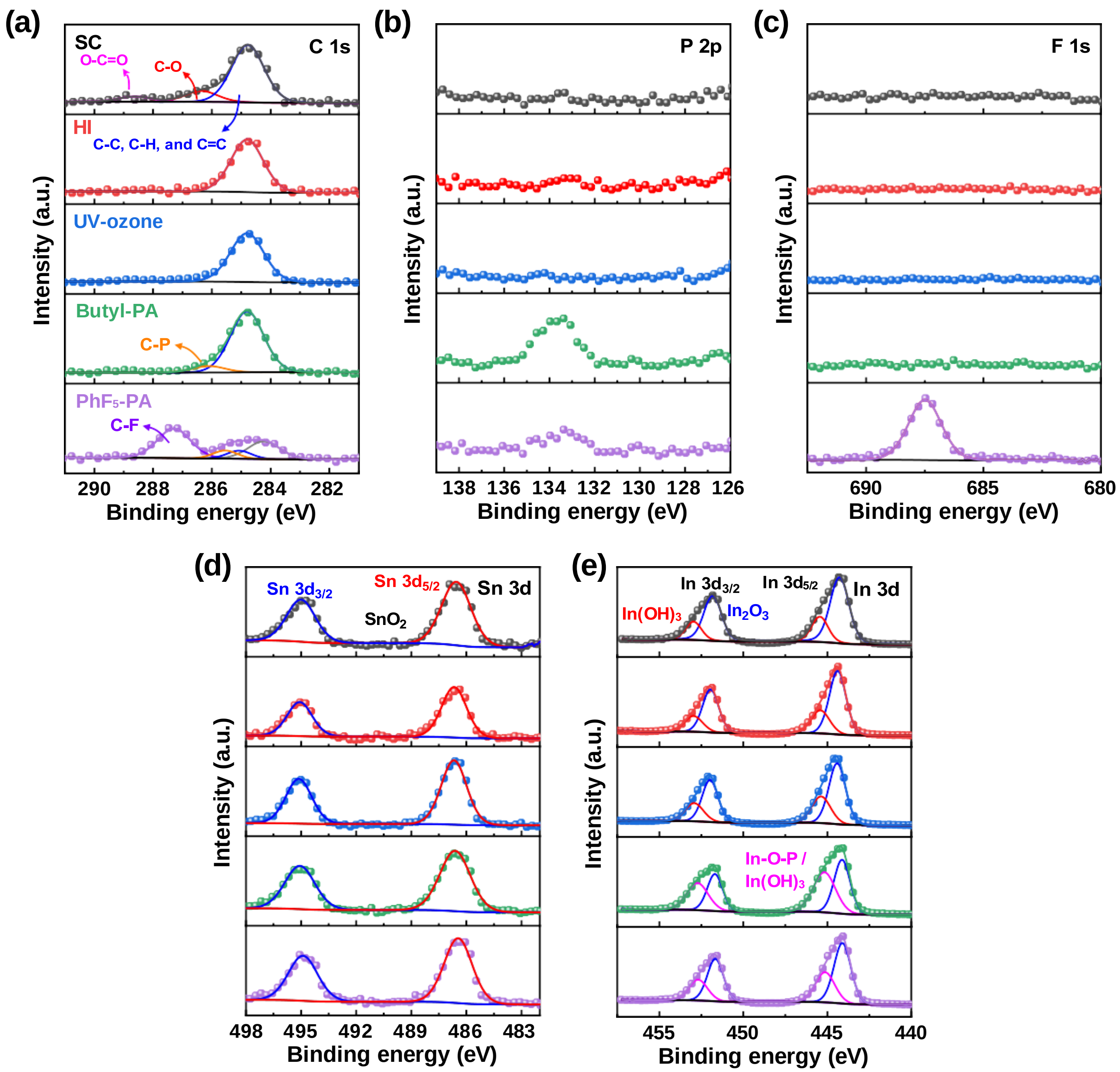

Figure S10. XPS spectra of (a) C 1s, (b) P 2p, (c) F 1s, (d) Sn 3d, and (e) In 3d core levels for surface-treated ITOs.

In Figure 2a, all substrates show evidence of the In_2O_3 peaks at around 530.0 eV in the O 1s core level spectra (**Table S4**).[3] For the solvent-cleaned ITO, C-O and O-C=O peaks at 532.3 and 533.4 eV, respectively, indicate that organic contaminants remain on the ITO surface, which are consistent with the high binding energy (BE) shoulders at 288.6 and 286.4 eV (**Figure S10a**)

in the C 1s core level spectrum.[3] The C-O-containing contaminants are represented as pink and red in Figure 2a. The UV-ozone treatment and HI etching can eliminate these contaminants with the formation of a more hydrophilic surface. Estimated hydroxide coverage on ITO surface was statistically higher in UV-ozone-treated ITO (23.1 %) than SC ITO and HI-etched ITO (17.0 and 18.9 %, respectively).[3] By using HI, we can easily control the etch of ITO layers in order to expose a metal oxide-rich surface, which is characterized by a near-surface Sn/In atomic ratio (0.07 ± 0.01) (versus 0.11 ± 0.01 for the other ITO substrates) (**Table S5**). C 1s core levels coming from the C-P and C-F bonds within the phosphonic acid modifiers demonstrate strong shifts to higher BEs in the C 1s spectra, while P=O and P-OH peaks in the P 2p core level spectra indicate an effective reaction and coverage between the PA-SAMs and the ITO substrate (**Figure S10a,b**).[4]

Table S4. Deconvolution parameters of XPS spectra of O 1s and C 1s core levels.

Treatments	O 1s (BE (eV), FWHM (eV), Area%)		C 1s (BE (eV), FWHM (eV), Area%)	
SC	In_2O_3	529.9, 1.20, 42.8	C-C, C-H, and C=C	284.8, 1.30, 77.7
	O_{vac}	530.9, 1.10, 16.6	C-O	286.4, 1.30, 14.8
	$In(OH)_3$	531.7, 1.20, 17.0	O-C=O	288.6, 1.30, 7.5
	C-O	532.3, 1.20, 17.3		
	O-C=O and H_2O_{ad}	533.4, 1.20, 6.3		
HI	In_2O_3	530.0, 1.13, 57.3	C-C, C-H, and C=C	284.8, 1.30, 100
	O_{vac}	530.8, 1.10, 17.0		
	$In(OH)_3$	531.5, 1.18, 18.9		
	C-O	532.5, 1.20, 6.7		
UV-ozone	In_2O_3	530.0, 1.15, 53.5	C-C, C-H, and C=C	284.8, 1.35, 100
	O_{vac}	530.8, 1.00, 14.9		
	$In(OH)_3$	531.5, 1.20, 23.1		
	C-O	532.5, 1.20, 8.6		
Butyl-PA	In_2O_3	529.8, 1.13, 35.6	C-C, C-H, and C=C	284.8, 1.32, 90.6
	O_{vac}	530.8, 1.00, 17.6	C-P	286.1, 1.30, 9.4
	P=O	531.6, 1.20, 33.7		
	P-OH	532.9, 1.20, 13.2		
PhF_5-PA	In_2O_3	529.7, 1.20, 47.3	Unassigned	284.2, 1.28, 29.3
	O_{vac}	530.7, 1.00, 16.4	C=C	285.1, 0.97, 10.1
	P=O	531.4, 1.20, 27.0	C-P	285.5, 0.98, 10.1
	P-OH	532.5, 1.20, 9.3	C-F	287.3, 1.34, 50.5

Table S5. Atomic concentrations of treated ITO surfaces obtained from XPS spectra.

Treatments	O	C	P	F	Sn	In
**SC**	44.00 ± 1.48 (2.21 ± 0.07)	31.91 ± 2.21 (1.47 ± 0.10)			2.39 ± 0.18 (0.11 ± 0.01)	21.70 ± 0.17 (1.00 ± 0.01)
**HI**	41.16 ± 1.25 (1.40 ± 0.04)	27.32 ± 2.04 (0.93 ± 0.07)			2.07 ± 0.17 (0.07 ± 0.01)	29.45 ± 0.15 (1.00 ± 0.01)
**UV-ozone**	43.73 ± 0.74 (1.55 ± 0.03)	24.92 ± 1.50 (0.89 ± 0.05)			3.21 ± 0.13 (0.11 ± 0.00)	28.14 ± 0.14 (1.00 ± 0.01)
**Butyl-PA**	46.17 ± 0.54 (2.21 ± 0.03)	24.30 ± 1.72 (1.16 ± 0.08)	6.31 ± 0.68 (0.30 ± 0.03)		2.37 ± 0.15 (0.11 ± 0.01)	20.86 ± 0.15 (1.00 ± 0.01)
**PhF_5-PA**	41.69 ± 1.06 (1.86 ± 0.05)	19.87 ± 1.42 (0.88 ± 0.06)	2.09 ± 0.73 (0.09 ± 0.03)	11.34 ± 0.39 (0.50 ± 0.02)	2.55 ± 0.16 (0.11 ± 0.01)	22.46 ± 0.14 (1.00 ± 0.01)

Parenthesis indicates relative atomic ratio to In (X/In)

7 Supplementary Note 6: Evidence of electrolyte penetration in photocathodes

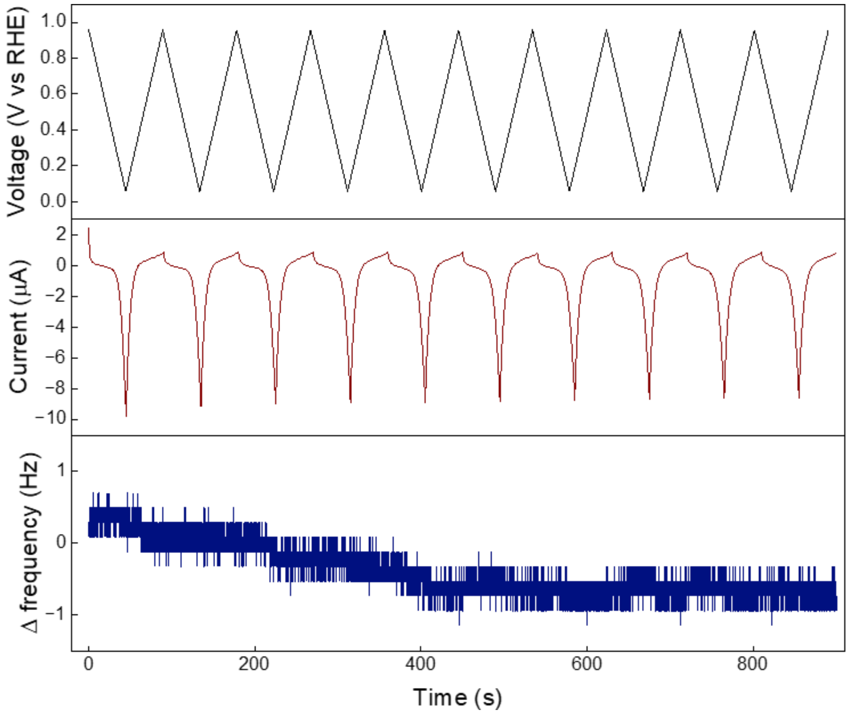

Figure S11. Time-dependent EQCM profiles of applied voltage, measured current, and frequency change for a BHJ film during CV scans at a scan rate of 20 mV s^{-1} in aqueous 0.1 M H_2SO_4 electrolyte.

We performed EQCM measurement on BHJ films to investigate the swelling behavior of hydrophobic conjugated polymers under applied bias in an aqueous electrolyte (Figure S11). We selected a potential window where no significant electrochemical reactions occurred, as evidenced by low currents (< 10 μA). During the scans, the mass increased over the initial 400 s before reaching saturation. Based on a polymer density of 1.1 g cm^{-3}, the calculated maximum swelling fraction (Δm/mass of BHJ film) is 0.29 %. This value is consistent with the hydrophobic nature of PTB7-Th:P(NDI2OD-T2) blend and the absence of electrochemical doping of the polymers.

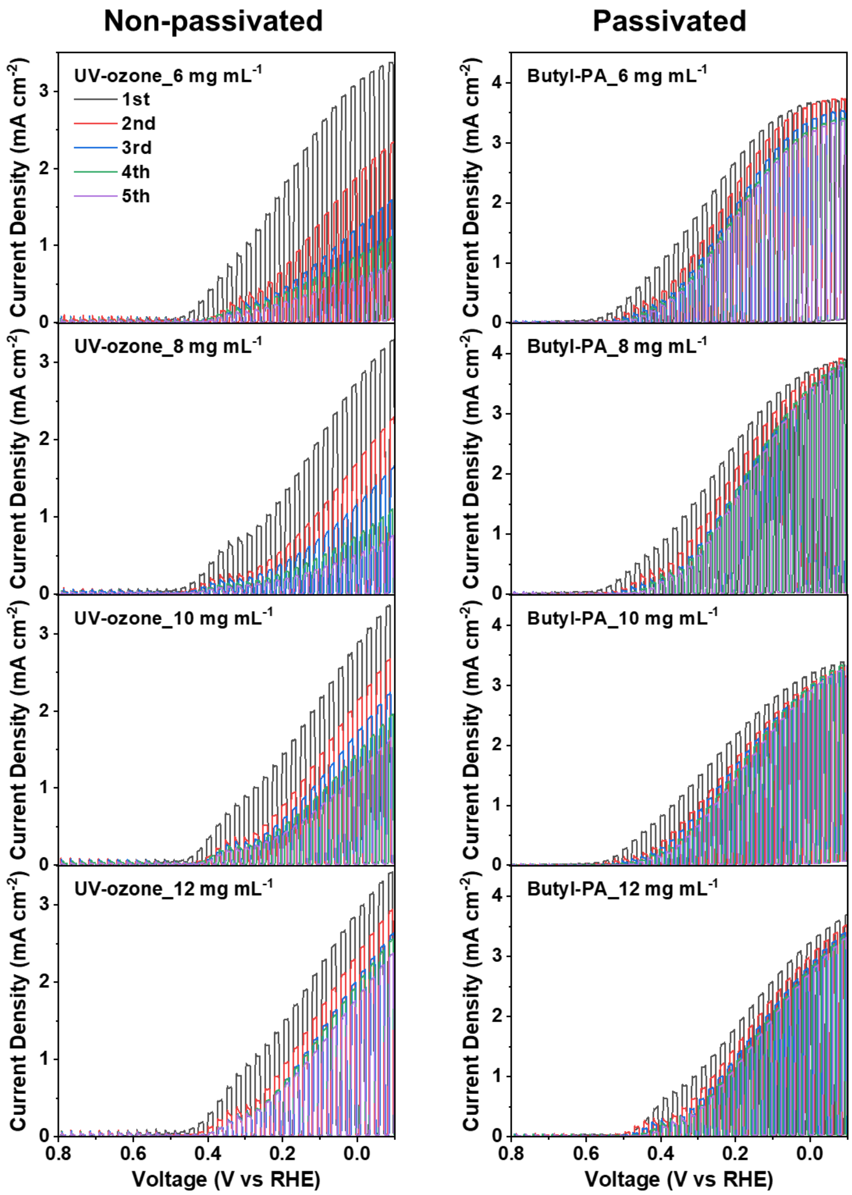

Figure S12. *J*-*V* curves of (a-d) UV-ozone and (e-h) Butyl-PA photocathodes of which BHJ layers were deposited by solution concentrations of 6, 8, 10, and 12 mg mL^{-1}, respectively.

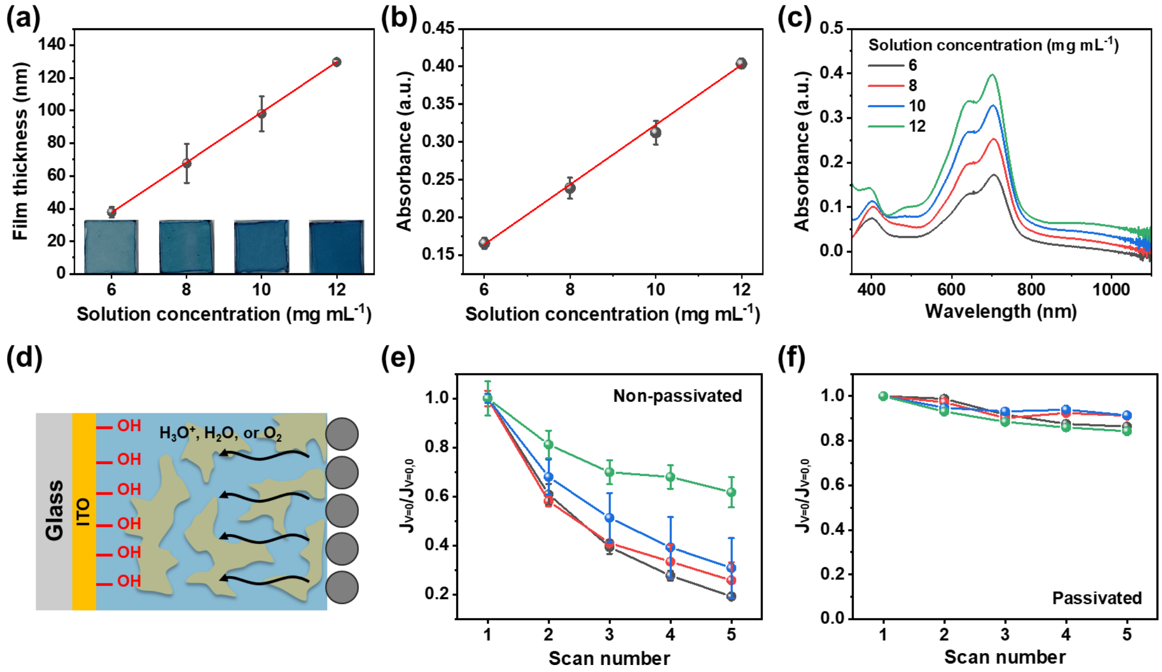

Figure S13. (a) BHJ layer thicknesses and (b) maximum absorbances as a function of BHJ solution concentration. Inset in (a) is photos of ITO/BHJ. (c) UV-vis absorption spectra of ITO/BHJ coated by different solution concentrations. (d) A scheme illustrating thickness-dependent electrolyte diffusion into buried ITO contact. Relative $J_{V=0}$ of (e) non-passivated (UV-ozone) and (f) passivated (Butyl-PA) photocathodes as a function of LSV scan number.

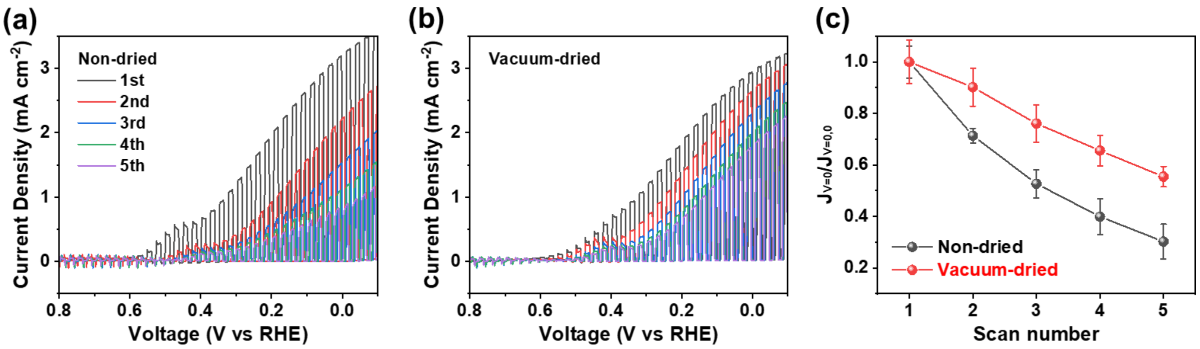

Figure S14. *J*-*V* curves of (a) control and (b) vacuum-dried non-passivated ITO contacts (UV-ozone) photocathodes in aqueous 0.1 M H_2SO_4 electrolyte. (c) Relative $J_{V=0}$ of the photocathodes as a function of LSV scan number.

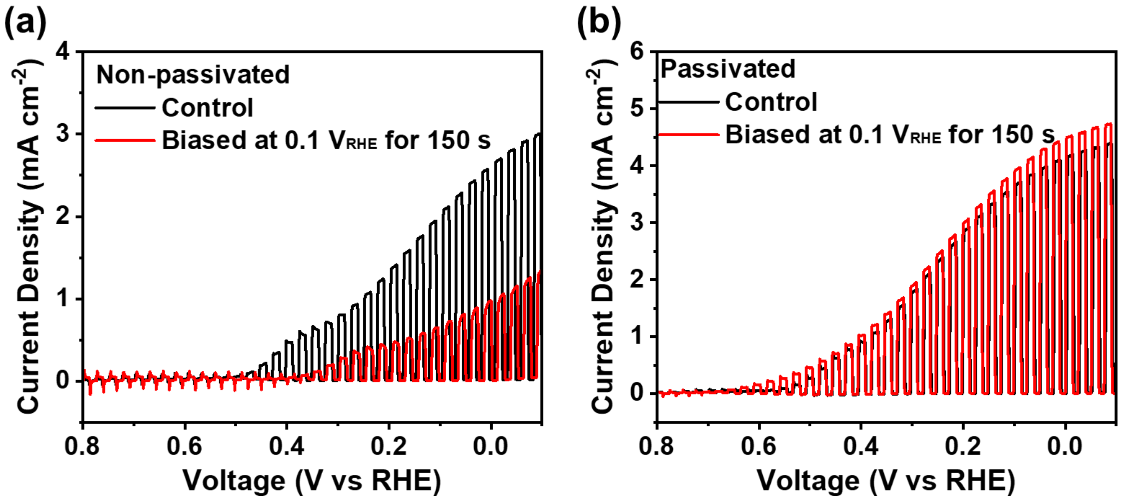

Figure S15. *J*-*V* curves of control and biased (a) non-passivated (UV-ozone) and (b) passivated (Butyl-PA) photocathodes at 0.1 V_{RHE} for 150 s in aqueous 0.1 M H_2SO_4 electrolyte under dark condition.

8 Supplementary Note 7: Time-dependent carrier recombination

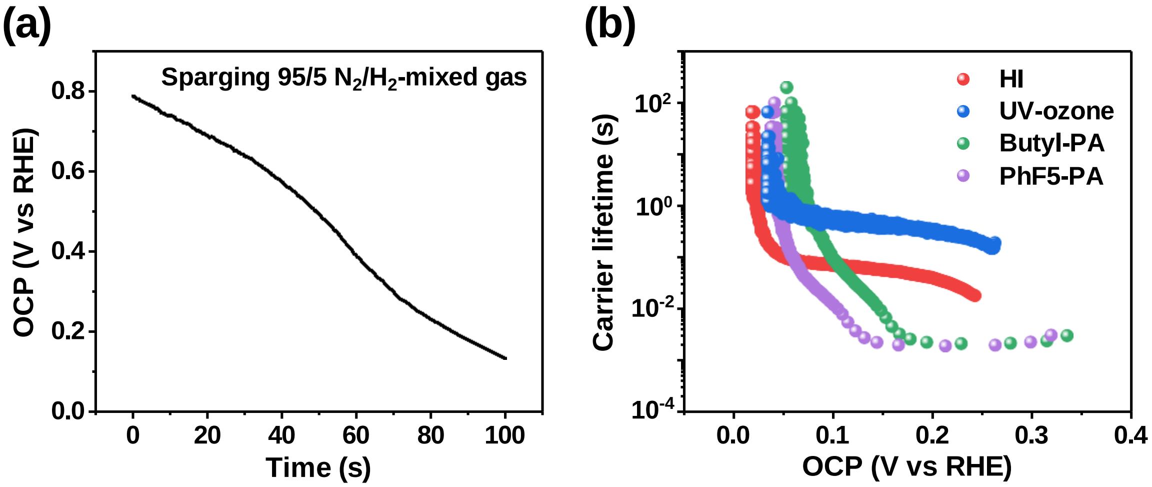

Figure S16. (a) OCP curve of a photocathode as a function of measurement time during 95/5 N_2/H_2-mixed gas sparging into an electrochemical cell. (b) Carrier lifetime as a function of OCP for HI, UV-ozone, Butyl-PA, and PhF_5-PA photocathodes extracted from transient OCP decay at 15 s.

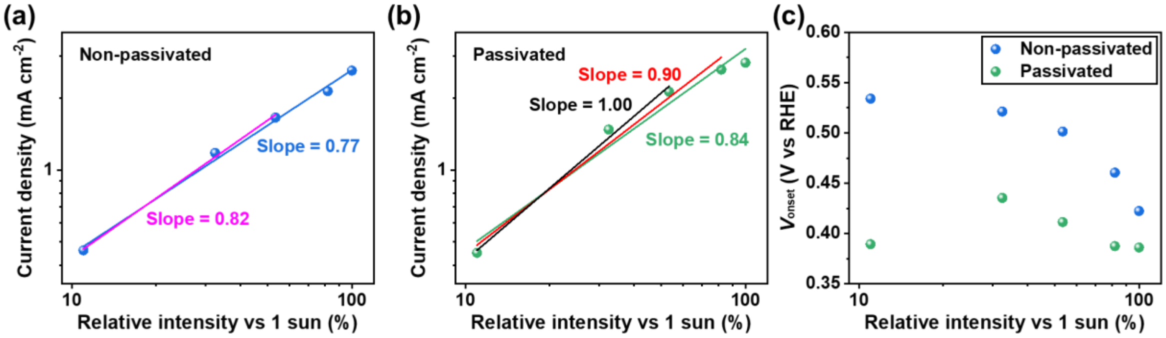

Figure S17. Light intensity-dependent $J_{V=0}$ of (a) non-passivated (UV-ozone) and (b) passivated (Butyl-PA) photocathodes (ITO/BHJ/Pt). (c) Light intensity-dependent V_{onset} of UV-ozone and Butyl-PA photocathodes. Non-linearity under 82% and 100% light intensities of 1 sun illumination are assumed to originate from the formation and adsorption of H_2 bubbles. The bubble-limited photocurrents underestimate the slope of Butyl-PA photocathode, as shown by the different fits that reduce the weight from the higher light intensities (more bubbles).

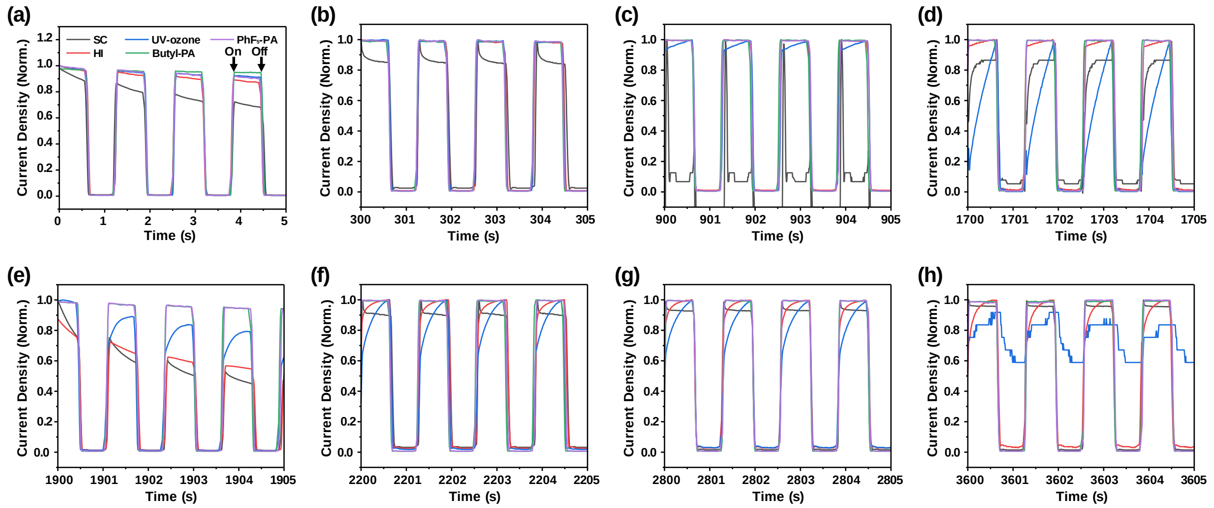

Figure S18. Chronoamperometry of soft photocathodes under chopped 1 sun illumination before electrolyte refilling at (a) 0–5, (b) 300–305, (c) 900–905, and (d) 1700–1705 s and after electrolyte refilling at (e) 1900–1905, (f) 2200–2205, (g) 2800–2805, and (h) 3600–3605 s. All photocurrents are normalized by local maximum photocurrents.

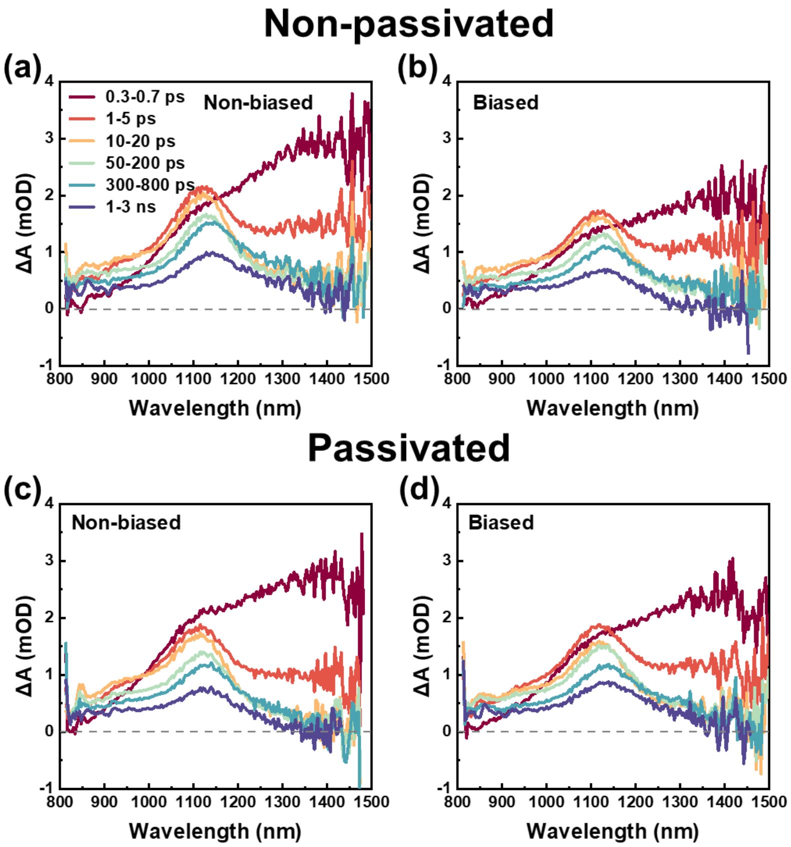

Figure S19. Transient absorption spectra of (a,b) non-passivated (UV-ozone) and (c,d) passivated (Butyl-PA) photocathodes (a,c) before and (b,d) after five LSV scans in aqueous 0.1 M H_2SO_4 electrolyte. The photocathodes were pumped at 650 nm (fluence = 5.76 µW) and probed at 1100 nm.

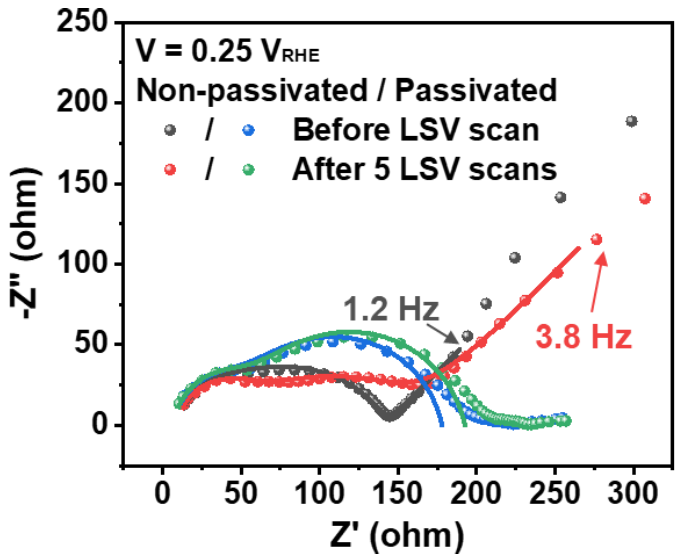

Figure S20. Nyquist plots of UV-ozone-treated and Butyl-PA-treated photocathodes before and after five LSV scans in aqueous 0.1 M H_2SO_4 electrolyte under 1 sun illumination.

Nyquist plots from electrochemical impedance data in **Figure S20** study the interfaces of the UV-ozone-treated and Butyl-PA-treated photocathodes at 0.25 V_{RHE} under 1 sun illumination and confirm that more photocarriers are accumulated at the polymer-electrolyte interphase and/or at the buried ITO/BHJ junction within UV-ozone-treated photocathode.[5-6] The Nyquist plots of UV-ozone and Butyl-PA photocathodes shows two merged semi-circles at high and moderate frequencies which are attributed to space charge layers at the polymer-electrolyte interphase and slow charge transfer at the ITO/BHJ interface, respectively. We extracted fitting parameters from the Nyquist plots (**Figure S21 and Table S6**). A Warburg element was added to fit Nyquist plots of UV-ozone photocathodes at low frequencies, indicating mass transport. At high frequencies, resistances and capacitances of both photocathodes were negligibly changed by LSV scans, which indicates BHJ layers were not damaged by electrochemical bias. At moderate frequencies, C_{CT} of UV-ozone photocathode increased an order of magnitude from 7.51 ± 1.01 to 45.35 ± 6.60 nF cm^{-2} after five LSV scans while those of Butyl-PA photocathode were almost preserved from 0.80 ± 0.36 to 1.07 ± 0.22 nF cm^{-2}.

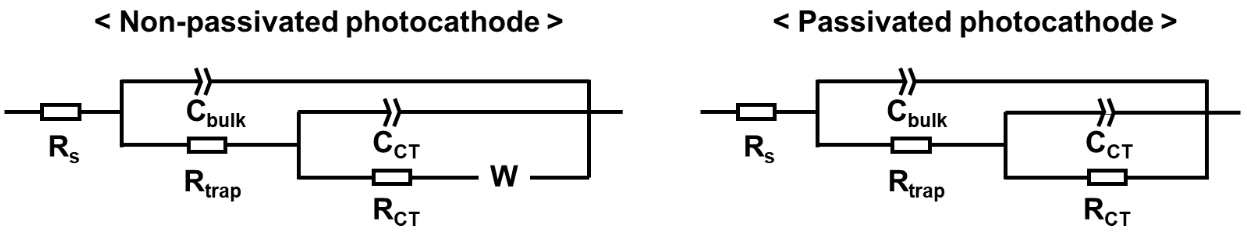

Figure S21. Equivalent circuits for photocathodes with non-passivated (UV-ozone) and passivated (Butyl-PA-treated) ITO electrodes.

Table S6. Fitting parameters of Nyquist plots of UV-ozone and Butyl-PA photocathodes using equivalent circuits in **Figure S21**.

		R_s (ohm cm^2)	R_{trap} (ohm cm^2)	C_{bulk} (nF cm^{-2})	R_{CT} (ohm cm^2)	C_{CT} (μF cm^{-2})	a	W (ohm s$^{-1/2}$)
**UV-ozone**	**Before LSV scans**	7.4 ± 0.3	32.2 ± 1.6	22.3 ± 0.7	55.9 ± 1.9	7.5 ± 1.0	0.67	107.5 ± 2.5
	**After LSV scans**	7.0 ± 0.3	32.2 ± 0.9	22.7 ± 0.7	71.9 ± 2.1	45.4 ± 6.6	0.57	541.4 ± 11.2
**Butyl-PA**	**Before LSV scans**	5.7 ± 0.2	40.4 ± 1.5	19.7 ± 0.5	73.5 ± 3.5	0.8 ± 0.4	0.84	
	**After LSV scans**	5.5 ± 0.2	42.1 ± 0.9	21.4 ± 0.5	81.9 ± 2.2	1.1 ± 0.2	0.84	

R_s Series resistance

R_{trap} Bulk trap resistance (1 MHz – 97 kHz)

C_{bulk} Bulk capacitance (1 MHz – 97 kHz)

R_{CT} Charge transfer resistance (97 kHz – 100 Hz)

C_{CT} Charge transfer capacitance (97 kHz – 100 Hz)

a Constant phase element

W Warburg coefficient (< 100 Hz)

9 Supplemental Note 8: FcOH/FcOH$^+$ redox chemistry on hole-only devices in the dark

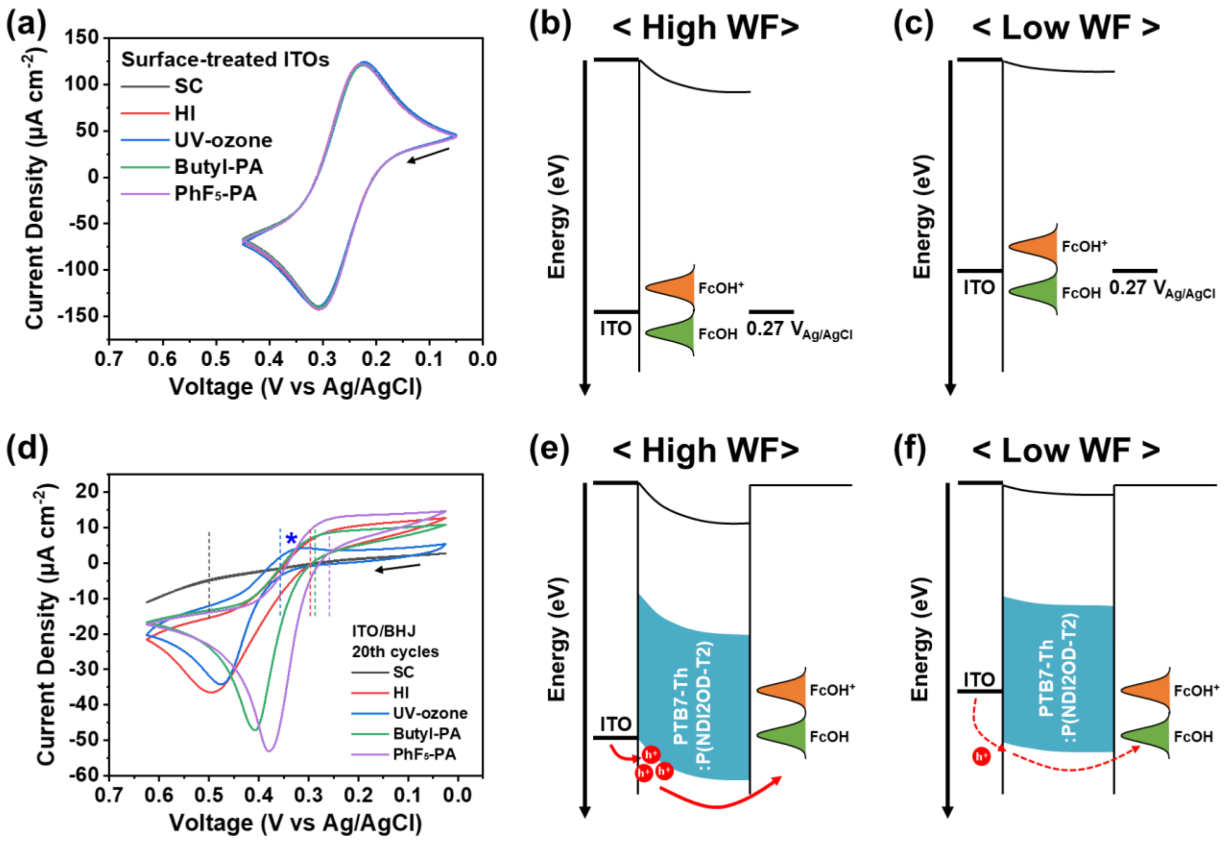

Figure S22. (a) CV curves of surface-treated ITO substrates in aqueous FcOH-rich electrolyte (1 mM FcOH + 0.1 M H_2SO_4) and (b,c) their energy level diagrams. (d) First CV curves of soft photocathodes (ITO/BHJ) and (e,f) their energy level diagrams. Dashed lines in (d) indicate onset potentials for oxidation reactions.

For hole transfer characterization, we performed CV scans for surface-treated ITO substrates and their photocathodes (ITO/BHJ). FcOH was selected as an electron donating redox couple due to its redox potential (0.27 $V_{Ag/AgCl}$) was lower than oxidation onset potentials of PTB7-Th (1.07 $V_{Ag/AgCl}$) and P(NDI2OD-T2) (1.28 $V_{Ag/AgCl}$), which suppresses direct polaron formation of bulk polymers. On surface-treated ITO substrates without BHJ layers, the Fermi levels of the ITO substrate and the redox probe are assumed to be pinned in **Figure S22a**. The CV curves overlap also indicates that the surface treatments do not change the diffusion coefficients and reaction kinetics of FcOH/FcOH$^+$ at ITO substrates. When we add the BHJ to the system, the redox

behavior of FcOH/$FcOH^+$ changes considerably. On semiconductor materials, electrochemical processes are dependent on the available density of states (DOS) that overlaps with the DOS of the redox probe considering the reorganization energy, diffusion coefficient, and reaction kinetics of the probe based on the Marcus-Gerischer model.[7-8] CP electrodes specifically undergo no band bending because they swell and get filled with electrolyte.[9-10] As stated in the main text, the standard potential of the FcOH/$FcOH^+$ probe is located between the HOMO and LUMO levels of PTB7-Th and P(NDI2OD-T2). This means there should be no DOS overlap between the BHJ and the probe, which prohibits direct charge transfer.

Small currents of ITO/BHJ photocathodes (< ± 2.0 μA cm^{-2}) in aqueous 0.1 M H_2SO_4 electrolyte support no polaron formation at least within 0.0–0.4 $V_{Ag/AgCl}$ (**Figure S22d**). However, the photocathodes showed high FcOH oxidation currents (10.2–46.4 μA cm^{-2}) in quasi-equilibrium states (20th cycles) except for SC photocathode. The FcOH oxidation currents and peak potentials were dependent on the Fermi levels of ITO contacts in the photocathodes. All these results suggest band bending at ITO-polymer contacts formed energy states near the redox potential, which dominates FcOH redox reaction on the photocathodes (**Figure S22e,f**). Low Fermi level of ITO contact in SC photocathode only had negligible DOS overlapping to FcOH/$FcOH^+$. Decreased peak currents on the photocathodes compared to on ITOs indicate the oxidation reaction was limited by slow diffusion of FcOH and/or slow kinetics. Overpotentials of FcOH oxidation reaction (> 0.08 V) on the photocathodes support the limited currents.

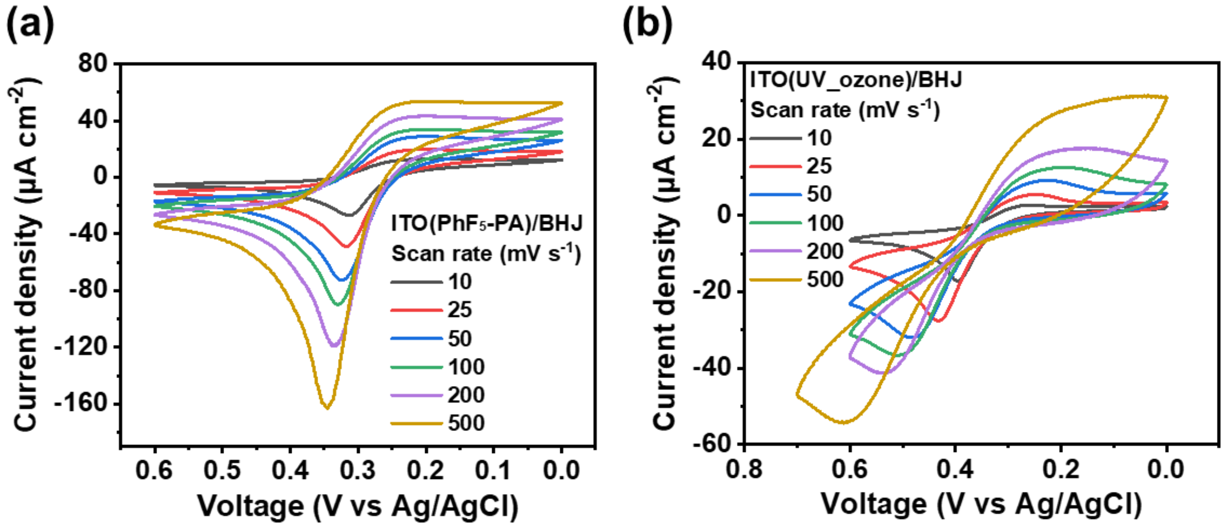

Figure S23. CV scans of (a) PhF_5-PA and (b) UV-ozone photocathodes (ITO/BHJ) at different scan rates in aqueous 1 mM FcOH + 0.1 M H_2SO_4 electrolyte (20th cycles).

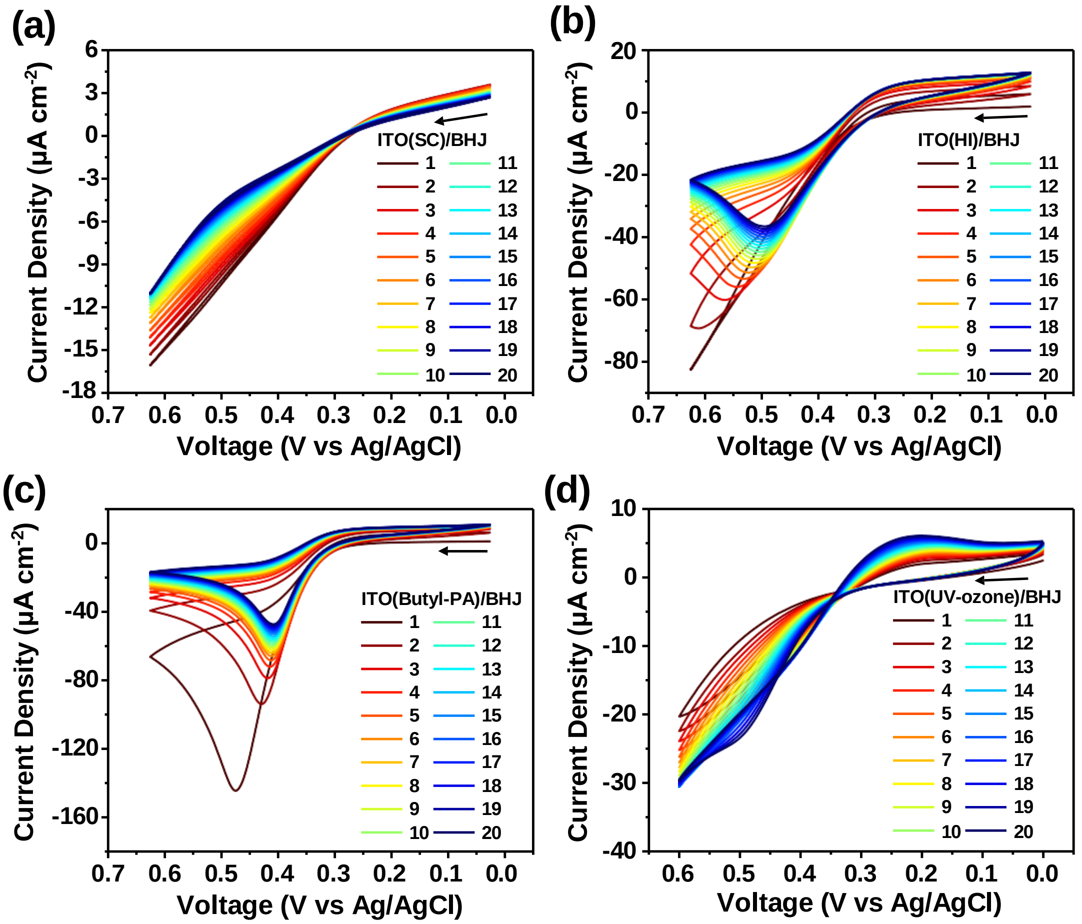

Figure S24. CV curves of (a) SC, (b) HI, (c) Butyl-PA, and (d) UV-ozone photocathodes (ITO/BHJ) in a FcOH-added electrolyte (1 mM FcOH + 0.1 M H_2SO_4). CV curves of UV-ozone photocathodes in (d) were obtained from another experimental batch.

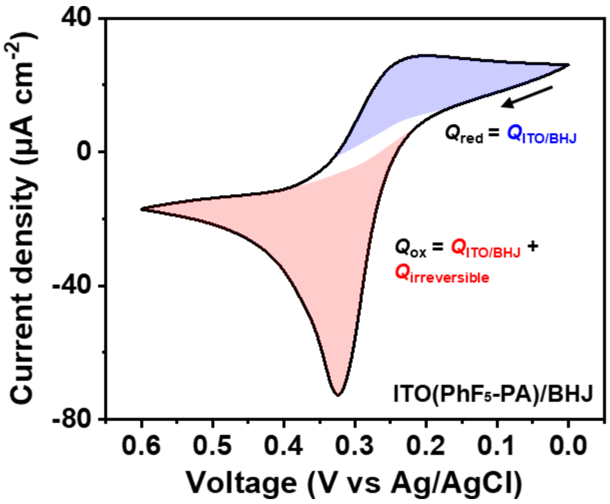

Figure S25. An example of the integration of Faradaic FcOH redox reactions.

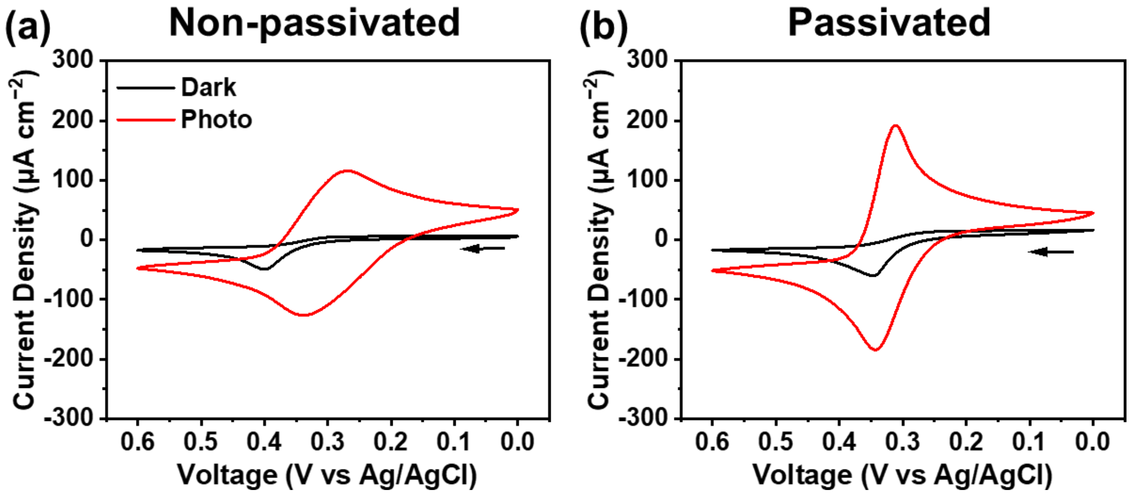

Figure S26. Increased reversibility of reactions due to increased photoconductivity in (a) non-passivated (UV-ozone) and (b) passivated (Butyl-PA) photocathodes in 1 mM FcOH + 0.1 M H_2SO_4 electrolyte under 1 sun illumination.

10 Supporting References

1. De Keersmaecker, M.; Tirado, J.; Armstrong, N. R.; Ratcliff, E. L., Defect Quantification in Metal Halide Perovskites Anticipates Photoluminescence and Photovoltaic Performance. *ACS Energy Lett.* **2024,** *9* (1), 243-252.
2. Cardona, C. M.; Li, W.; Kaifer, A. E.; Stockdale, D.; Bazan, G. C., Electrochemical considerations for determining absolute frontier orbital energy levels of conjugated polymers for solar cell applications. *Adv. Mater.* **2011,** *23* (20), 2367-2371.
3. Donley, C.; Dunphy, D.; Paine, D.; Carter, C.; Nebesny, K.; Lee, P.; Alloway, D.; Armstrong, N. R., Characterization of Indium-Tin Oxide Interfaces Using X-ray Photoelectron Spectroscopy and Redox Proccesses of a Chemisorbed Probe Molecule: Effect of Surface Pretreatment Conditions. *Langmuir* **2002,** *18*, 450-457.
4. Tang, C. S.; Antoni, M.; Schönbächler, I.; Keller, B.; Textor, M.; Vörös, J., Electrically-Assisted Formation and Desorption of Dedecyl Phosphate Self-Assembled Monolayers on Indium Tin Oxide Surfaces. *ECS Trans.* **2006,** *1* (28), 29-43.
5. Cho, H.-H.; Yao, L.; Yum, J.-H.; Liu, Y.; Boudoire, F.; Wells, R. A.; Guijarro, N.; Sekar, A.; Sivula, K., A semiconducting polymer bulk heterojunction photoanode for solar water oxidation. *Nat. Catal.* **2021,** *4* (5), 431-438.
6. Yao, L.; Guijarro, N.; Boudoire, F.; Liu, Y.; Rahmanudin, A.; Wells, R. A.; Sekar, A.; Cho, H. H.; Yum, J. H.; Le Formal, F.; Sivula, K., Establishing Stability in Organic Semiconductor Photocathodes for Solar Hydrogen Production. *J. Am. Chem. Soc.* **2020,** *142* (17), 7795-7802.
7. Rudolph, M.; Ratcliff, E. L., Normal and inverted regimes of charge transfer controlled by density of states at polymer electrodes. *Nat. Commun.* **2017,** *8* (1), 1048.
8. Neelamraju, B.; Rudolph, M.; Ratcliff, E. L., Controlling the Kinetics of Charge Transfer at Conductive Polymer/Liquid Interfaces through Microstructure. *J. Phys. Chem. C* **2018,** *122* (37), 21210-21215.
9. Bisquert, J.; Garcia-Belmonte, G.; Garcia-Canadas, J., Effects of the Gaussian energy dispersion on the statistics of polarons and bipolarons in conducting polymers. *J. Chem. Phys.* **2004,** *120* (14), 6726-6733.
10. Garcia-Belmonte, G.; Vakarin, E. V.; Bisquert, J.; Badiali, J. P., Doping-induced broadening of the hole density-of-states in conducting polymers. *Electrochimica Acta* **2010,** *55* (21), 6123-6127.
